# Symmetric Ligand Binding Pathways and Dual-State Bottleneck in [NiFe] Hydrogenases from Unbiased Molecular Dynamics

**DOI:** 10.1021/acs.jpclett.5c01673

**Published:** 2025-07-29

**Authors:** Farzin Sohraby, Ariane Nunes-Alves

**Affiliations:** Institute of Chemistry, 26524 Technische Universität Berlin, Straße des 17. Juni 135, 10623 Berlin, Germany

## Abstract

[NiFe] hydrogenases make up a family of enzymes that can be used to produce biofuel, thus making them important for industrial applications. In this work, we utilized unbiased molecular dynamics simulations to capture binding and unbinding events of the substrate, H_2_, to and from the [NiFe] hydrogenases from two different organisms. We obtained multiple (un)­binding events and reproduced experimental association rate constants. We observed symmetry between the binding and unbinding pathways used by H_2_ to access and leave the catalytic site. Moreover, we found that the main bottleneck for ligand binding, the distance between residues V74 and L122, can shift between two states with different bottleneck widths, a feature which can be exploited to modulate the access of small molecules to the catalytic site. The pathway probabilities presented here can be used to benchmark enhanced sampling methods which investigate protein–ligand binding.

## Graphical abstract

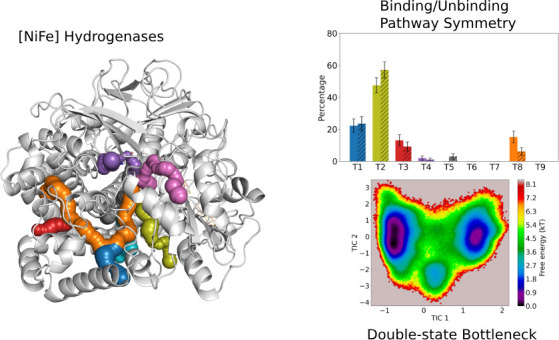

It has been established in recent years^1–4^ that binding kinetic rates should be taken into consideration in drug design efforts, since in some cases residence times can have a strong correlation with drug efficacy.^4–7^ This has led to the development of many computational methods which can be used to predict binding kinetic rates for protein–ligand complexes and investigate the associated (un)­binding paths.^8–11^ Kinetic rates are nonequilibrium properties, and their values depend on the paths for ligand binding and unbinding. Therefore, a fundamental aspect of understanding and predicting ligand binding kinetics is the characterization of binding and unbinding paths and associated probabilities. Knowledge of binding pathways can also be explored in biotechnology and enzyme engineering. For instance, mutant enzymes can be rationally designed to block or promote access of substrate or inhibitor molecules to the catalytic site.^12–15^

Investigating protein–ligand binding using unbiased molecular dynamics (UMD) simulations is challenging, because the time scales for the occurrence of (un)­binding events are usually longer in comparison to the ones achieved by UMD simulations.^16,17^ Microsecond-long UMD simulations were performed by D. E. Shaw research to investigate the binding of small molecule inhibitors to Src kinase and G protein-coupled receptors (GPCRs).^18,19^ Observing binding events with UMD simulations was an important achievement, but only a few events could be captured, preventing sound estimation of kinetic rates or pathway probabilities. Additionally, very few people have access to computing clusters like Anton,^20,21^ used to perform such UMD simulations. In recent years, researchers started to combine MD simulations with methods to enhance sampling,^22–29^ such as τ-Random Acceleration Molecular Dynamics (τRAMD)^30^ or metadynamics,^31^ to obtain a large number of unbinding events, which enable them to estimate kinetic rates and pathway probabilities. While computed kinetic rates can be compared to experimental ones to benchmark new enhanced sampling methods, there is no clear data set or reference to benchmark the observed pathways and associated probabilities. In previous reviews,^8,9^ we showed that authors using different enhanced sampling methods obtain different unbinding paths for systems such as T4 lysozyme, kinases, and trypsin. For T4 lysozyme, reasonable predictions of the kinetic rates can be obtained as long as the main pathway is sampled.^9^ However, a complete understanding of binding paths with high and low probabilities can enable the rational design of mutant proteins with new or modified paths to either block or facilitate ligand binding, as done before for haloalkane dehalogenase and ABCG transporters.^14,32–34^

In this work, we propose as a data set to benchmark the pathway probabilities obtained from enhanced sampling methods the pathway probabilities for binding and unbinding of H_2_ to and from two different [NiFe] hydrogenases, the hydrogenase from *Desulfovibrio fructosovorans* (Df hydrogenase) and the hydrogenase from *Megalodesulfovibrio gigas* (Mdg hydrogenase), obtained from a total of 18.75 μs of UMD simulations (75 replicas of 250 ns) for each hydrogenase. Df hydrogenase and Mdg hydrogenase are both O_2_-sensitive hydrogenases and they have low sequence identity, 65.5%, despite the fact that the two enzymes have an almost identical secondary structure (Figure 1). We chose hydrogenase as a model system because for small gas molecules such as H_2_ the binding rates are fast, and it is feasible to capture a reasonable number of (un)­binding events using UMD simulations. Additionally, data analysis of the UMD simulations revealed that there are two states for the bottleneck identified by us and others^13,35–38^ as one of the major factors which regulates ligand binding in Df hydrogenase, the distance between residues at positions 74 and 122 in the large subunit of Df hydrogenase (Figure 1). This dual-state bottleneck mechanism could be exploited to engineer mutants of hydrogenases that are resistant to inhibitors such as CO and O_2_.

**Figure 1:**
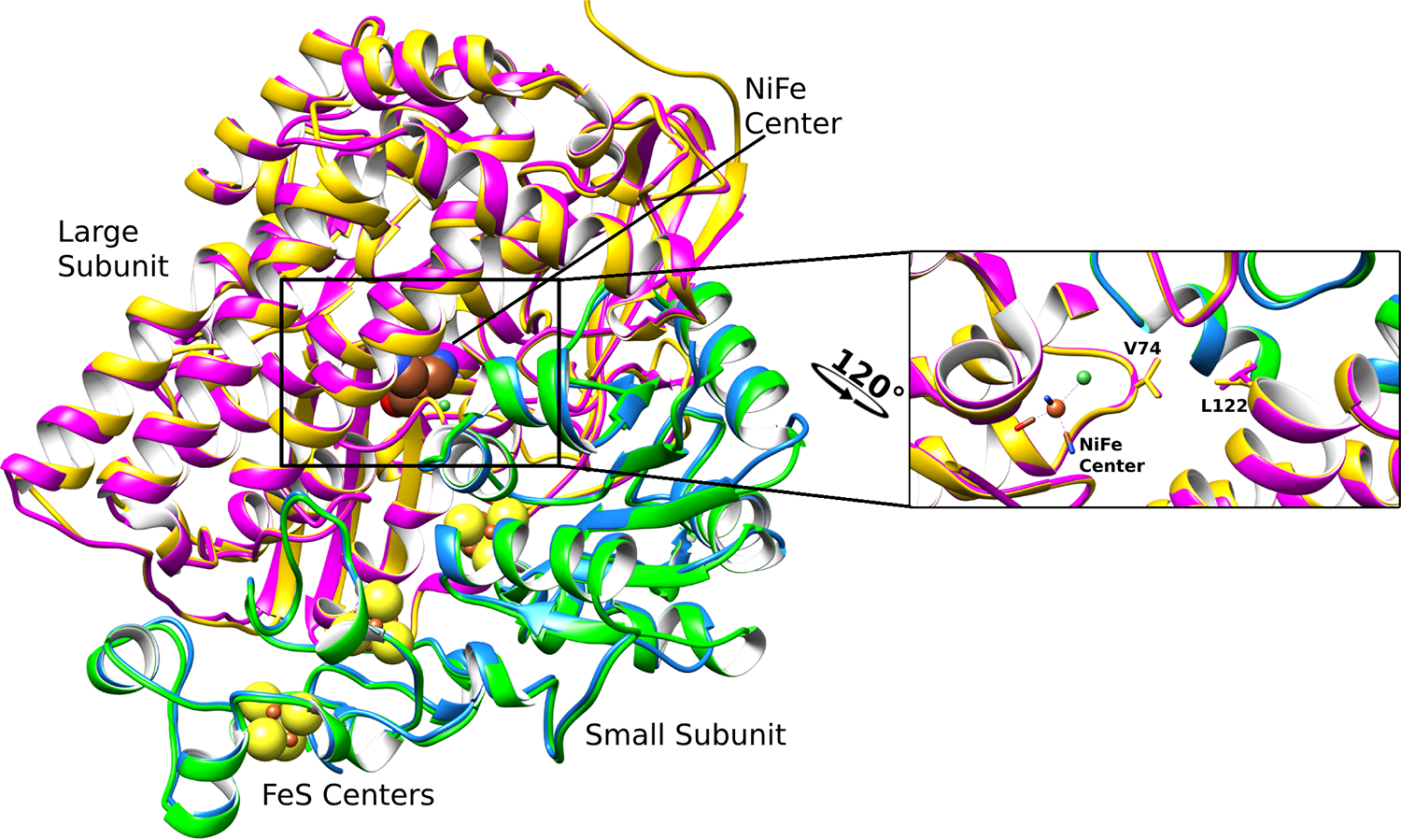
Aligned crystallographic structures of Df hydrogenase and Mdg hydrogenase (PDB ID 1YQW^39^ and 1YQ9,^39^ respectively) show the high similarity of the secondary structures of the two hydrogenases. The small subunits of Df hydrogenase and Mdg hydrogenase are shown in blue and green, and the large subunits are in yellow and magenta, respectively. The catalytic site and the position of the residues of the bottleneck for ligand binding, V74 and L122, are shown in the inset.

We put 100 H_2_ molecules in the simulation box randomly and let the gas molecules diffuse independently, explore the tunnels inside the enzymes, and eventually reach the hydrogenase active site, the [NiFe] center. Since we are using a conventional force field, no covalent bond is formed between the [NiFe] center and H_2_ once it approaches the active site. The bound state was achieved when H_2_ reached a distance of 5 Å or lower from the center of mass of the [NiFe] center, and it was also near the interface of the Ni and Fe atoms of the [NiFe] center. The unbound state was achieved when H_2_ was fully solvated and displayed no contact with any of the atoms of the enzyme (a contact was formed when the atom–atom distances were below 4 Å).

Using UMD simulations, we could obtain 43 and 100 binding events of H_2_ to Df hydrogenase and Mdg hydrogenase, respectively, and 41 and 99 unbinding events of H_2_ from Df hydrogenase and Mdg hydrogenase, respectively (). For Df hydrogenase, experimentally measured Michaelis constant (K_m_) and catalytic constant (*k*_cat_) values for H_2_ were reported by Liebgott et al.,^40^ leading to an experimental *k*_on_ value calculated to be 1.9 × 10^6^ M^–1^·s^–1^ (see equations in ). With 100 H_2_ molecules in the simulation box, leading to a concentration of 119.7 mM, and a mean first passage time (FPT) of 89.6 ± 69.2 ns, calculated from the binding events from UMD simulations, the computational *k*_on_ was calculated to be 9.3 ± 1.2 × 10^7^ M^–1^·s^–1^, which is in close agreement to the experimental *k*_on_ value. The computational *k*_off_ value was calculated to be 2.2 ± 0.3 × 10^8^ s^–1^, but there is no experimental *k*_off_ value reported for comparison.

We performed the same UMD simulations for the inhibitors O_2_ and CO binding to Df hydrogenase, as we did for the substrate H_2_. However, the number of (un)­binding events obtained was low, 4 and 2 binding and 4 and 1 unbinding events for O_2_ and CO, respectively, even with double the amount of simulation time for CO (37.5 μs). The pathways for O_2_ and CO can be found in .

We mapped the tunnels for gas diffusion in the crystallographic structures of both Df and Mdg hydrogenase using CAVER 3.0 (Figure 2) to later map the tunnels to the (un)­binding events identified in UMD simulations, following our previous works.^13,35^ We found that the tunnels are similar for the different enzymes except for the fact that T9 in Df hydrogenase is not present in Mdg hydrogenase. There are also changes in tunnels T3 and T8 in the Mdg hydrogenase, which have common parts with T1, in contrast to tunnels T3 and T8 in Df hydrogenase, which are independent of T1. The binding and unbinding events obtained from UMD for Df and Mdg hydrogenase were identified following the definitions of bound and unbound states above and manually assigned to the tunnels identified (Figure 3, ). The assignment was based on matching of entry (for binding events) and exit points (for unbinding events) between (un)­binding events and tunnels. We use the term “pathway” to refer to a tunnel used for (un)­binding in the UMD simulations.

**Figure 2:**
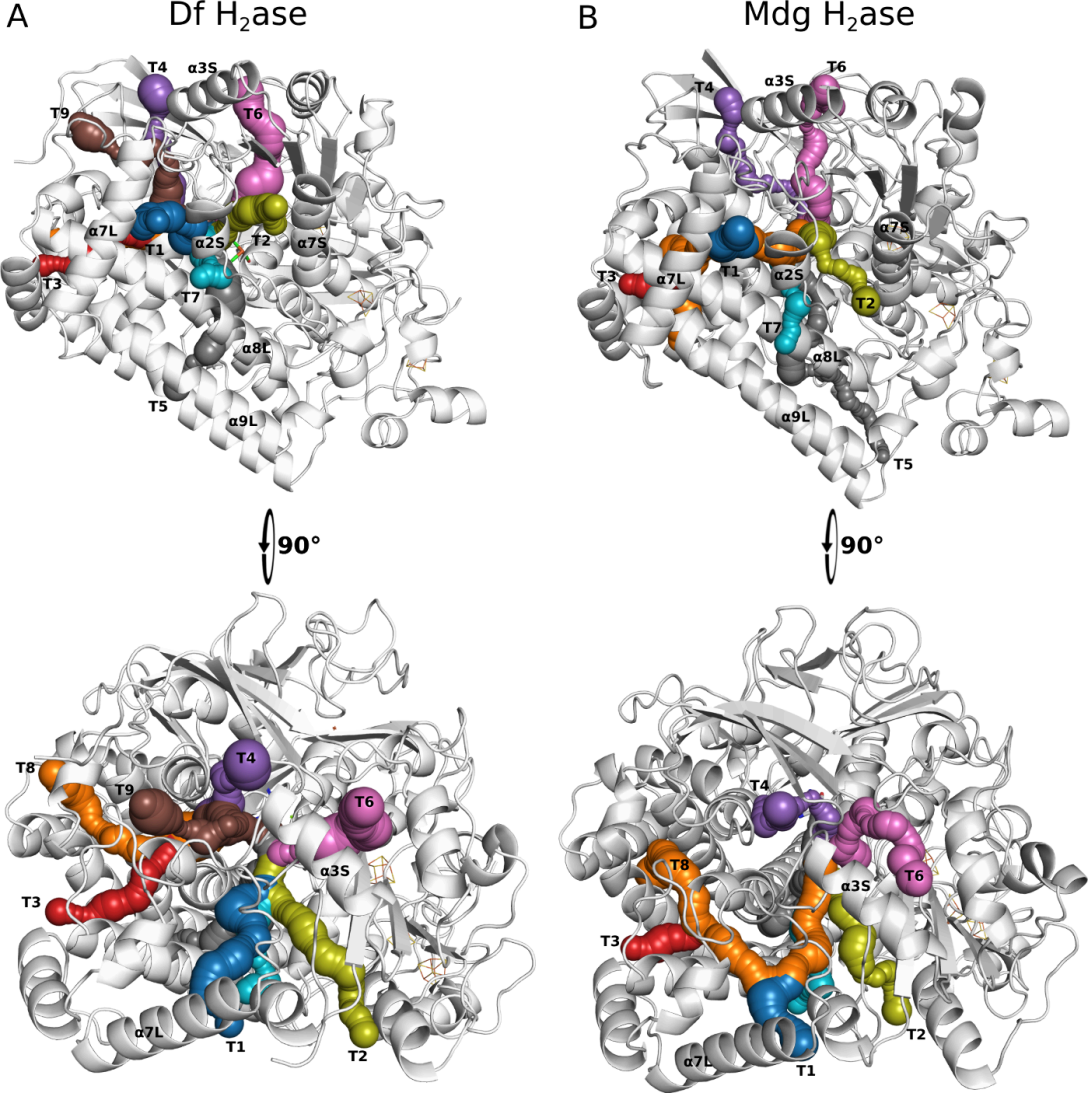
Tunnels identified in the crystallographic structures of A) Df hydrogenase (Df H_2_ase, PDB ID 1YWQ^39^) and B) Mdg hydrogenase (Mdg H_2_ase, PDB ID 1YQ9 ^39^) using the CAVER 3.0 plugin in Pymol.^41,42^ Nine tunnels (T1-T9) and eight tunnels (T1-T8) were identified in Df and Mdg hydrogenase, respectively. The secondary structures are named according to order of appearance in the primary structure and subunits (S for small and L for large). Example: α2S, second α-helix from the small subunit.

**Figure 3:**
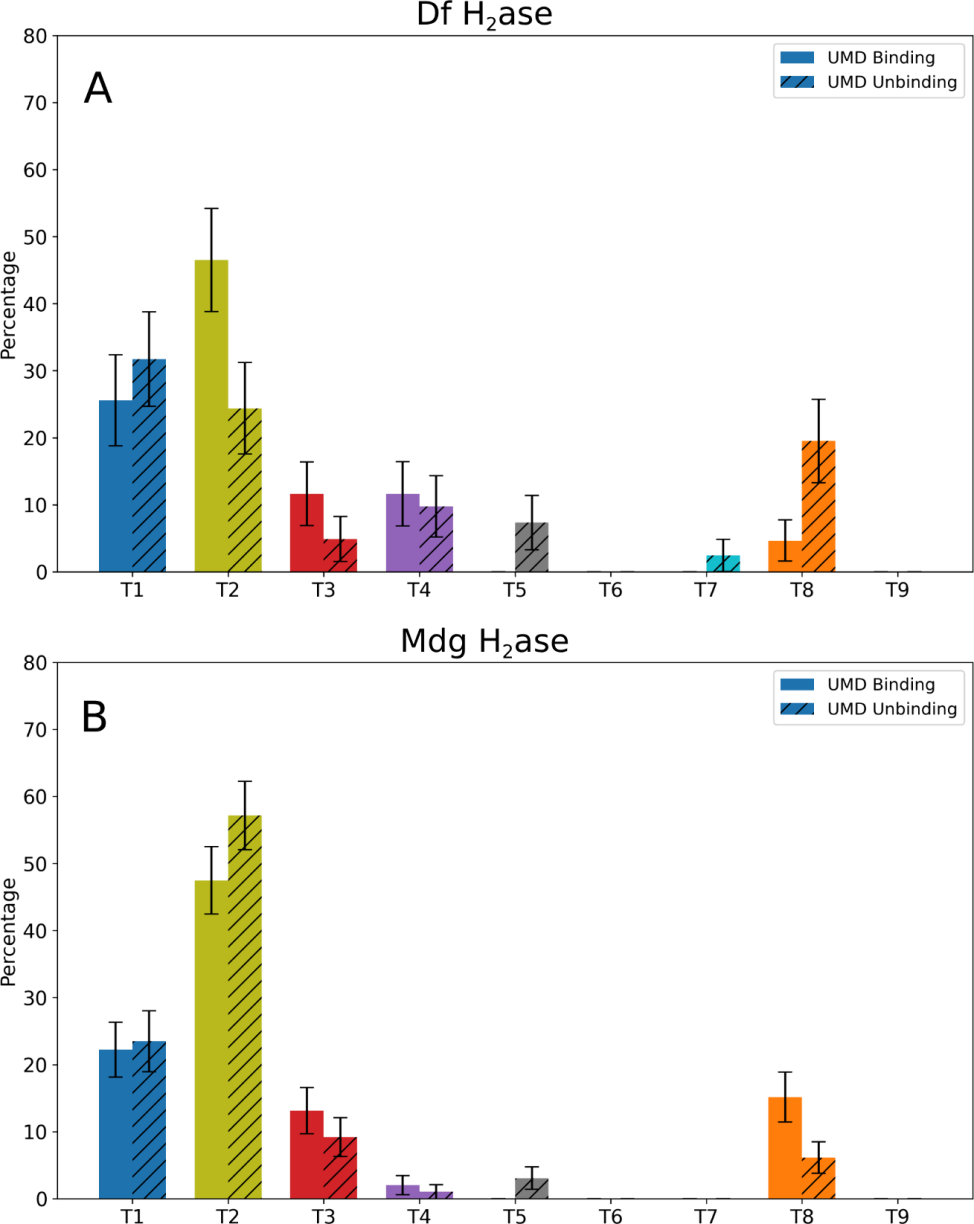
Pathway probabilities are symmetric for H_2_ binding and unbinding in A) Df hydrogenase (Df H_2_ase) and B) Mdg hydrogenase (Mdg H_2_ase). The colors of each pathway match the colors of the associated tunnels in Figure 2. The standard error for each bar comes from bootstrapping. The p-values obtained from the chi-square test were 0.053 and 0.121 for Df and Mdg hydrogenase, respectively, indicating that there is no significant difference between the binding and unbinding pathway probabilities (considering a threshold of 0.05).

For the two enzymes, pathways T1 and T2 were the most probable paths for binding and unbinding (Figure 3), in overall agreement with our previous works, where we used τRAMD to investigate unbinding of H_2_, CO, and O_2_ from Df hydrogenase and Mdg hydrogenase.^13,35^ Pathways T1 and T2 are similar, sharing the region close to the [NiFe] center (Figure 2, bottom panels) and bifurcating close to helix α2S. It is also notable that some tunnels, like T6 and T9, were not used for (un)­binding events.

Next, we tested whether there was symmetry of the binding and unbinding pathways for the two hydrogenases or, in other words, if the pathway probabilities for binding and unbinding were the same. Pathway symmetry is expected for equilibrium processes, based on the principle of detailed balance or microscopic reversibility proposed by Boltzmann.^43,61^ A chi-square test was conducted, and the p-values obtained were 0.053 and 0.121 for Df hydrogenase and Mdg hydrogenase, respectively (Tables S5 and S6), which indicates that the differences in pathway usage between binding and unbinding events in each hydrogenase are not statistically significant (using a p-value of 0.05 as a threshold). The p-value for Df hydrogenase is near the threshold, and this can be traced to the differences in the populations of paths T5 and T8 (). Paths T5 and T8 have a lower probability in binding events. Such differences can be the result of a limited number of (un)­binding events.

In previous works,^13,35^ we used τRAMD to simulate unbinding of H_2_, CO, and O_2_ from Df and Mdg hydrogenases. τRAMD is an enhanced sampling technique which applies a force of constant magnitude and random orientation on the center of mass of the ligand to facilitate ligand unbinding. The relative residence times obtained with τRAMD, from tens of unbinding events for one protein–ligand complex, can be compared with experimental values for benchmarking and can be used to rank multiple ligands or different mutants of a protein. We performed τRAMD simulations for 10 different mutants of Df hydrogenase, using a force with a magnitude of 1 kcal/(mol·Å) and 75 unbinding events to estimate relative residence times, and we could successfully reproduce the ranking of absolute residence times measured experimentally (R = 0.79, ρ = 0.75).

We compared the unbinding pathway probabilities obtained from our previous works using τRAMD^13,35^ with the pathway probabilities obtained here using UMD and found that they are significantly different according to the chi-square test. We obtained p-values of 0.013 and 1.7e^–15^ for Df hydrogenase and Mdg hydrogenase, respectively (, ). In Df hydrogenase, the differences can be mainly attributed to path T2 (), which has a lower probability in τRAMD. In Mdg hydrogenase, the differences can be attributed mostly to paths T2 and T5 (), which have lower and higher probability in τRAMD, respectively. For the case of Df hydrogenase, differences between the pathway probabilities could have arisen from the number of unbinding events, which is larger in τRAMD. In Df hydrogenase, UMD simulations have similar probabilities for paths T1 and T2, while in τRAMD the probabilities are higher for path T1. Since paths T1 and T2 are similar, they can potentially be grouped together, which would lead to more comparable probabilities. Differences may also arise from the longer UMD simulations (hundreds of nanoseconds), which may have captured long time scale dynamics not observed in short τRAMD simulations (tens of nanoseconds), and from the use of a force in τRAMD to speed up unbinding, which can potentially affect unbinding and the associated pathway probabilities, as observed before for ligand unbinding in GPCRs.^44^ Despite the differences, τRAMD was able to identify path T1 as one of the most probable paths for H_2_ unbinding from Df and Mdg hydrogenase.

Additionally, we performed data analysis to investigate the dynamics of the main bottleneck for ligand unbinding, located between two evolutionary conserved hydrophobic residues, V74 and L122 (Df hydrogenase sequence numbering), in the UMD simulations. The bottleneck distance was identified by us^13^ and others^37,38,40^ as one of the main factors modulating the residence times for CO bound to different mutants of Df hydrogenase. In our previous work,^13^ we identified a strong correlation between the distances between residues V74 and L122 and the experimentally measured residence times, suggesting that these two residues act as a bottleneck for gas transit to the catalytic site. Equilibrium properties such as free energy landscapes (FELs) should not be directly computed from an ensemble of short independent simulations. Therefore, we constructed a Markov state model^46^ (MSM; details in the Supporting Information in ) and used it to compute a reweighted FEL (Figure 4B). UMD simulations revealed that the bottleneck distances have two populated states in Df hydrogenase: open and closed (Figure 4). The minimum distance between residues V74 and L122 can range from ∼ 4 up to ∼ 10 Å (Figure 4). Part of this variation can be attributed to dihedral changes in L122 (Figure 4) and part to changes in the secondary structure in the region around residue L122, which switches between 3_10_-helix, extended strand and turn according to the DSSP analysis^45^ (). While the crystallographic structure is in the closed state, UMD simulations suggest that the open state is the most stable state for Df hydrogenase. In the τRAMD simulations from previous work,^13^ we only observed the open state of the bottleneck (). We also investigated the presence of the open and closed states in Mdg hydrogenase. In this case, while the FEL indicates the presence of two states, there are only minor structural changes in the regions including V74 and L122 (), and the range of distances sampled in the UMD simulations is narrower (Figure 4A), indicating that the bottleneck in Mdg hydrogenase is in the closed state. The kinetic diameter of gas molecules (H_2_, CO, and O_2_) ranges between 2.9 and 3.8 Å (). While these diameters are lower than the minimum width of the bottleneck in the two hydrogenases, it is expected that motions in the bottleneck can hinder or facilitate gas transit. Information about the dual-state bottleneck can be exploited to engineer mutant enzymes that block the passage of larger gas molecules, such as CO and O_2_, which inhibit some hydrogenases. It remains to be tested whether the dual-state bottleneck is part of the dynamics of other hydrogenases, contributing to the regulation of residence times and access of the gas molecules to the catalytic site.

**Figure 4:**
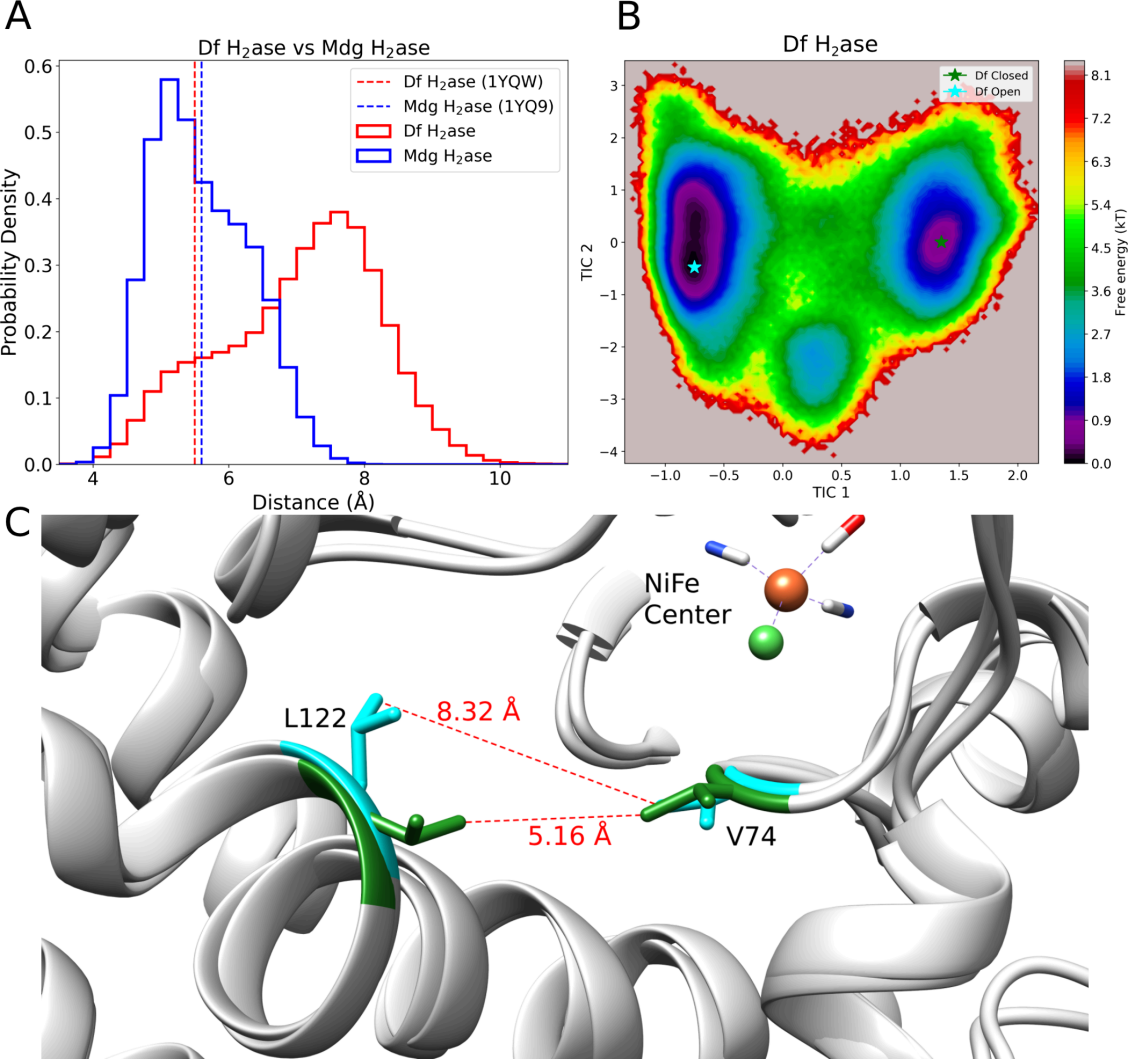
Dual-state bottleneck in Df hydrogenase (Df H_2_ase) observed in unbiased molecular dynamics simulations. A) Probability density of lowest distance values between residues V74 and L122 in Df hydrogenase and Mdg hydrogenase (Mdg Df H_2_ase). The distance values in the crystallographic structures are shown as traced lines. B) Free energy landscape of the V74-L122 bottleneck in Df hydrogenase computed using a Markov state model (details in the ). Two main states were identified: open and closed. C) Representative snapshots of Df hydrogenase in the open (cyan residues) and closed (green residues) states of the V74-L122 bottleneck. The location of the snapshots in the free energy landscape in panel B is indicated with green and cyan stars.

It has long been believed that the mechanism of tolerance in [NiFe] hydrogenases is due to the proximal and distal FeS centers, which are located in the small subunit and can reduce the [NiFe] center following an O_2_ or a CO attack. However, recent experiments using chimeric forms of *Escherichia coli* [NiFe] hydrogenases^47^ found that sensitivity to the inhibitor CO can be set by the large subunit and that specific structural determinants in both subunits contribute to inhibitor tolerance in the enzymes. This is evidence that structural information about the binding pathways and associated bottlenecks is an important factor in the tolerance mechanism. Recently, Grinter et al.^48^ revealed the energy extraction mechanism of the O_2_-tolerant Huc hydrogenase from *Mycobacterium smegmatis* using experiments and MD simulations. The authors obtained H_2_ and O_2_ (un)­binding events using UMD simulations and found that only H_2_ could enter the binding site, while O_2_ was sterically excluded by a series of bottlenecks present in the tunnels. O_2_ could reach the active site only in mutants where the bottlenecks were relieved. This is another evidence that the bottlenecks in the binding pathways can be exploited to achieve inhibitor-tolerance and optimized enzymes. Taking into consideration the mechanistic insights obtained here for Df hydrogenase, we propose introducing mutations in the region around the α-helix containing L122 to limit the motions of the residues in the bottleneck for ligand binding and lock the bottleneck in the closed state, restricting the access of inhibitors such as O_2_ and CO, which have diameters larger than those of H_2_, to the catalytic site. This may increase the tolerance of the enzyme to inhibitors.

In this work, we utilized UMD simulations for sampling binding and unbinding events of H_2_ to and from two different [NiFe] hydrogenases, Df hydrogenase and Mdg hydrogenase. The computed *k*_on_ value reproduced the experimental *k*_on_ value for the association of H_2_ to Df hydrogenase. We characterized path probabilities for binding and unbinding and found that there is symmetry between the binding and unbinding pathways for both enzymes. We also compared the path probabilities from this work using UMD simulations with the ones obtained in our previous works using τRAMD, and we found that τRAMD can identify the most probable pathways for H_2_ dissociation from hydrogenases. We expect this observation to be true for other systems, as long as large protein conformational changes are not required for ligand dissociation. Data analysis revealed that the main bottleneck that controls the access of ligands to the catalytic site can have two states in Df hydrogenase. The pathway populations obtained from UMD simulations can be used as a data set to benchmark enhanced sampling methods that aim to investigate ligand binding. Additionally, the mechanistic insights obtained for the gating of ligand access to the catalytic site of Df hydrogenase can lead to novel strategies to modulate gas diffusion inside hydrogenases.

## Computational Methods

The crystallographic structures of Df hydrogenase and Mdg hydrogenase (PDB IDs 1YQW^39^ and 1YQ9,^39^ respectively) were obtained from the Protein Data Bank.^49^ The force field bonded parameters and the partial charges of the metal centers were obtained from the works of Smith et al.^50^ and Teixeira et al.,^51^ respectively. The protonation states of the residues at pH 7, the pH used for measuring experimental kinetic rates,^40^ were determined using Propka version 3.5.2,^52–54^ as implemented in the program pdb2pqr version 2.1.1.^55,56^ The force field parameters of H_2_ (bonded parameters, Lennard-Jones parameters, and partial charges) were obtained from Wang et al.^57^ The protein was placed in the center of a cubic box with a distance of 1.5 nm from all edges, and then 100 H_2_ molecules were added to the box randomly (minimum distance of 1 nm from the hydrogenase) with a concentration of ∼ 120 mM. Finally, the system was solvated with the TIP3P^58^ water model. Then, sodium and chloride ions were added to produce an ionic strength of 118 mM, which was adopted to reproduce the conditions used for the protein film voltammetry experiments to obtain kinetic rates.^40^ All of the MD simulations were unbiased and were performed by GROMACS 2024.2 ^59^ and the Amber ff99SB^60^ force field. We kept the force field and, when possible, all of the parameters the same as the simulations performed previously with τRAMD for Df hydrogenase and Mdg hydrogenase^13,35^ to make a fair comparison between results from τRAMD simulations and UMD simulations. In total, we performed 75 replicas for each of the two hydrogenases, and each simulation had a duration of 250 ns, leading to a total simulation time of 18.75 μs for each hydrogenase. The MD simulations have been performed using two types of GPUs, Nvidia 1080 and 2080, on multiple nodes available at the High Performance Computer (HPC) of the Technical University of Berlin, and the performance for this system size, with 110,000 atoms, was roughly 40 ns/day on average. More details of the methods can be found in the .

## Supplementary Material





## Data Availability

The data to reproduce the UMD simulations (input files, starting structures and topologies) and the trajectories obtained are uploaded on Zenodo in 4 separate parts (‘10.5281/zenodo.15521025’, ‘10.5281/zenodo.15520947’, ‘10.5281/zenodo.15520978’, ‘10.5281/zenodo.15521025’); the code to analyze the data, the code for building and validating the MSM and the Pymol session files containing the Df and Mdg hydrogenase tunnels identified by CAVER can be found on Github: https://github.com/FarzinSohraby/SI-H2ase-UMD.

## Notes

1) Description of the methods used for the UMD simulations and for data analysis. 2) Equations to calculate *k*_on_ values using experimental data and mean first passage times (MPFTs) from UMD simulations. 3) Paths and MFPTs obtained from UMD simulations (Tables S1–S3). 4) Percentages of paths used for binding and unbinding in Df and Mdg hydrogenases (Table S4). 5) Contribution of pathway probabilities to the results of the chi-square tests (Tables S5–S8). 6) Comparison between pathway probabilities for unbinding obtained from τRAMD and UMD simulations (Figure S1 and S2). 7) Markov state model building and validation (Figures S3–S8). 8) DSSP analysis of the region around residue L122 in Df hydrogenase (Figure S9). 9) Comparison between probability densities of the bottleneck widths in τRAMD and UMD simulations (Figure S10). 10) Breck’s kinetic diameter of small gas molecules (Table S9). 11) Free energy profile, snapshots of representative structures and DSSP analysis of the residues in the V74-L122 bottleneck in Mdg hydrogenase (Figure S11). See also Data Availability Statement (PDF)
Transparent Peer Review report available (PDF)
