# Supplementary material for: Symmetric Ligand Binding Pathways and Dual-State Bottleneck in [NiFe] Hydrogenases from Unbiased Molecular Dynamics
Source: J Phys Chem Lett. 2025 Jul 29;16(31):7960–7. doi: 10.1021/acs.jpclett.5c01673 (PMC12337149; doi:10.1021/acs.jpclett.5c01673)
Supplement: Supplementary file 1 [file jz5c01673_si_001.pdf]

Supplementary Information

Symmetric Ligand Binding Pathways and Dual-State Bottleneck in [NiFe] Hydrogenases from Unbiased Molecular Dynamics

Farzin Sohraby[ǂ], Ariane Nunes-Alves[ǂ]*

[ǂ]Institute of Chemistry, Technische Universität Berlin, Straße des 17. Juni 135, 10623 Berlin, Germany

*Corresponding author: ferreira.nunes.alves@tu-berlin.de

Methods

System set up and molecular dynamics simulations

The crystallographic structures of the hydrogenase from *Desulfovibrio fructosovorans* (Df hydrogenase) and the hydrogenase from *Megalodesulfovibrio gigas* (Mdg hydrogenase) (PDB IDs 1YQW[1] and 1YQ9[1], respectively) were obtained from the Protein Data Bank[2]. The force field bonded parameters and the partial charges of the metal centers were obtained from the works of Smith et al.[3] and Teixeira et al.[4], respectively. The protonation states of the residues at pH 7, the pH used for measuring experimental kinetic rates[5], were determined using Propka version 3.5.2[6–8], as implemented in the program pdb2pqr version 2.1.1[9,10]. The force field parameters of H_2 (bonded parameters, Lennard-Jones parameters and partial charges) were obtained from Wang et al[11]. The protein was placed in the center of a cubic box with a distance of 1.5 nm from all edges, and then 100 H_2 molecules were added to the box randomly with a concentration of ~120 mM. Finally, the system was solvated with the TIP3P[12] water model. Then, sodium and chloride ions were added to produce an ionic strength of 118 mM, which was adopted to reproduce the conditions used for the protein film voltammetry experiments to obtain kinetic rates[5]. All of the molecular dynamics (MD) simulations were unbiased and performed using GROMACS 2024.2[13] and the Amber ff99SB[14] force field. We kept the force field and, when possible, all of the parameters the same as the simulations performed previously with τRAMD for Df hydrogenase and Mdg hydrogenase[15,16] to make an accurate comparison between

results from τRAMD and unbiased MD.

The starting structures were energy minimized using the steepest descent algorithm until the maximum force was less than 10 kJ.mol^{-1}.nm^{-1}. Then, the system was heated to 313 K using the Berendsen thermostat[17] in 4 steps of short simulations, starting from 10 K to 50, 150 and then 313 K, each with a duration of 100 ps. The MD simulations were performed at 313 K because the experiments to measure the Michaelis constant (K_m) for the reaction between H_2 and Df hydrogenase have been carried out at this temperature[5]. This K_m value was used to calculate the experimental k_{on} value. Next, the pressure was equilibrated to 1 bar using the Berendsen barostat[17] with a simulation time of 1000 ps. The equilibrium runs to adjust temperature and pressure were performed with positional restraints with a force constant of 1000 kJ.mol^{-1}.nm^{-2} on all heavy atoms. After temperature and pressure equilibration, additional steps were performed to reduce the positional restraints over the system's heavy atoms in 4 steps (force constants of 500, 200, 50 and 0 kJ.mol^{-1}.nm^{-2}). In all simulations, after equilibration, temperature and pressure coupling were achieved with the Nose-Hoover thermostat[18,19] and the Parrinello-Rahman barostat, respectively[20,21]. The covalent bonds to hydrogen atoms of the proteins were constrained using the Linear Constraint Solver (LINCS) algorithm[22]. Bond lengths in the solvent were constrained using the SETTLE algorithm[23]. The long-range electrostatic interactions were treated using the Particle Mesh Ewald (PME) method with a real-space cutoff of 1.2 nm, PME order of four, and a Fourier grid spacing of 1.2 Å[24,25]. Van der Waals forces were computed using a cutoff of 1.2 nm. All the simulations were performed using a time step of 2 fs. In total, we performed 75 replicas for each of the two hydrogenases. Each MD simulation had a duration time of 250 ns, leading to a total simulation time of 18.75 μs for each hydrogenase. The MD simulations have been performed using two types of GPUs, Nvidia 1080 and 2080, on multiple nodes available at the High Performance Computer (HPC) of the Technical University of Berlin, and the performance for this system size, with 110,000 atoms, was roughly 40 ns/day on average.

Data analysis

Analysis of trajectories and (un)binding events were performed using GROMACS 2024.2 utilities[13], MDAnalysis 2.4.3[27,28], MDTraj 1.9.7[29] and UCSF Chimera 1.17.3[30]. The distances of all 100 H_2 molecules to the [NiFe] center in all 75 replicas of each system were calculated

separately. The bound state was achieved when the gas molecule reached a distance of 5 Å or lower to the center of mass of the [NiFe] center, and it was located close to the interface of the Ni and Fe atoms in the [NiFe] center. The proximity to this interface is required for proper catalysis (in the case of the substrate, H_2) or enzyme inhibition (in the case of the inhibitors, O_2 and CO). The unbound state was achieved when the gas molecule was fully solvated and displayed no contacts with any of the atoms of the enzyme (a contact was formed when atom-atom distances were below 4 Å). The first passage time (FPT) for the unbinding events was defined as the time the gas molecule took going from the bound state to the unbound state, whereas the FPT for the binding events was defined as the time from the start of the simulation until the H_2 molecule reached the bound state.

We used CAVER 3.0 PyMOL Plugin[26] for tunnel identification in the crystallographic structures of Df hydrogenase and Mdg hydrogenase (PDB 1YQW[1] and 1YQ9[1], respectively). The coordinates of the center of mass of the [NiFe] center were used as the starting point. A minimum probe radius of 0.9 Å and a clustering threshold of 3.5 Å were used. Other settings were set to default values. These settings are the same settings used in our previous works for tunnel identification[15,16]. The pymol sessions showing the tunnels are available as supporting information on Github (https://github.com/FarzinSohraby/SI-H2ase-UMD).

The binding and unbinding events obtained from UMD simulations of Df and Mdg hydrogenase were identified following the definitions of bound and unbound states explained above. These events were then manually matched to the tunnels shown in Figure 3. The assignment was carried out by comparing the entry points (for binding) and exit points (for unbinding) of each event with the tunnels identified by CAVER.

Dimensionality reduction for the analysis of the bottleneck states and construction of Markov state models (MSM) was done by pyemma 2.5.2[31] using time-lagged independent component analysis (TICA) with a lag time of 1 ns. As features, we used the pairwise distances between all heavy atoms of the V74 and L122 residues during the entire unbiased molecular dynamics (UMD) simulations. The free energy landscapes were obtained using MSM, as described in the section "Markov state model construction and validation for UMD simulations of Df hydrogenase". The minimum distance between the CG1/CG2 atom in V74 and CD1/CD2 atom in L122 residues in each frame was used for the probability density analysis.

Calculation of the kinetic rates

For validating the kinetic rates achieved by UMD simulations, we calculated the experimental k_{on} using the data for H_2 binding to Df hydrogenase published by Liebgott et al.[5] For H_2 the Michaelis constant (K_m) was available in units of matm (H_2), and we converted it to units of mol/L using the ideal gas law equation.

$$K_{\mathrm{m}}(mol/L) = \frac{n}{V} = \frac{P}{RT} \quad (1)$$

- n is the number of moles (in mol), V is the volume (in L), P is the pressure (in this case, 10 matm (H_2)), R is the ideal gas constant (0.0821 L.atm/mol.K) and T is the temperature (313 K).

Then, the converted K_m (0.00041 mol/L) and the experimental k_{cat} value (760 s^{-1}) were used in the following equations to calculate k_{on}.

$$K_{\mathrm{m}} = \frac{k_{\mathrm{off}} + k_{cat}}{k_{on}} \quad (2)$$

For hydrogenases[5,32], $k_{off} << k_{cat}$

$$k_{\mathrm{on}} \sim \frac{k_{cat}}{K_m} \quad (3)$$

We obtained an experimental k_{on} value of $1.9 * 10^6$ M^{-1}. s^{-1}.

The computational k_{on} was calculated using the equation below, where [L] is the H_2 concentration in the system (119.7 mM):

$$k_{\mathrm{on\ comp}} = \frac{1}{\mathrm{MFPT} \times [\mathrm{L}]} \quad (4)$$

Mean First Passage Time (MFPT) is the average of FPTs for the binding events captured in the simulations (Tables S1 and S2). We obtained a MFPT value equal to 89.6 ± 69.2 ns in the

UMD simulations. The computational k_{on} value was calculated to be $9.3 * 10^7 \pm 1.2 * 10^7$ M^{-1}. s^{-1}. The standard error was obtained by bootstrapping the FPTs values using 10000 bootstrap samples.

For the calculation of the computed k_{off} values, we used the average of the FPTs of the unbinding events in the simulations, which are equivalent to average residence time (RT) values. The RT values were converted to k_{off} values using the following equation:

$$k_{\text{off comp}} = \frac{1}{RT} \tag{5}$$

We obtained a RT value of 4.6 ± 4.3 ns in the UMD simulations. The computational k_{off} value was calculated to be $2.2 * 10^8 \pm 0.3 * 10^8$ s^{-1}, but there is no experimental k_{off} value reported for comparison. The standard error was obtained by bootstrapping the FPTs values using 10000 bootstrap samples.

Mean first passage times computed from simulations

Tables S1 and S2 show FPTs obtained and tunnels used in the binding and unbinding events of H_2 captured in the UMD simulations of Df hydrogenase and Mdg hydrogenase.

Table S1. H_2 binding and unbinding events captured in 75 independent UMD simulations (250 ns length) of Df hydrogenase.

Replica #	Tunnels in[a]	First Passage Time - Binding[b] (ps)	Tunnels out[c]	Time of the unbound state[d] (ps)	First Passage Time - Unbinding[e] (ps)
1	T1	44340	T2	48310	3970
7	T4	154950	T4	177380	22430
9	T2	19340	T8	20850	1510
9	T8	41200	T8	44430	3230
9	T4	226860	NA[f]	NA[f]	NA[f]
11	T2	64970	T2	66490	1520
11	T1	12800	T1	16200	3400
11	T1	171490	T1	179750	8260
14	T8	27250	T7	33070	5820
14	T4	87170	T8	88750	1580
14	T4	35250	T4	37880	2630
17	T3	232090	T1	233500	1410

20	T2	121320	T1	131910	10590
21	T3	45290	T5	56010	10720
23	T1	243710	NA[f]	NA[f]	NA[f]
25	T2	19750	T1	25520	5770
29	T2	3040	T3	3950	910
31	T1	4780	T1	6440	1660
32	T2	38850	T1	40520	1670
34	T1	176330	T5	178410	2080
35	T3	164400	T1	166790	2390
35	T2	165440	T1	168700	3260
36	T2	101770	T2	104130	2360
36	T4	143650	T4	159950	16300
37	T2	55010	T2	61940	6930
44	T1	145470	T1	147210	1740
47	T3	5490	T8	7300	1810
47	T1	222270	T1	231940	9670
49	T2	103750	T2	107440	3690
49	T3	86660	T8	89770	3110
49	T2	18770	T2	24030	5260
52	T2	158550	T8	160810	2260
56	T2	117710	T1	119070	1360
56	T2	112270	T1	115050	2780
56	T2	81710	T2	84980	3270
56	T1	78220	T3	80070	1850
63	T2	27370	T2	30720	3350
65	T2	5110	T8	9570	4460
66	T2	30730	T8	39740	9010
67	T1	51170	T4	52130	960
68	T2	72800	T2	76800	4000
73	T2	77690	T5	86290	8600
75	T1	57890	T2	62980	5090

a) “Tunnel in” refers to the tunnel that the H_2 molecule used for binding.
b) “First Passage Time - Binding (ps)” refers to the time in the simulation when the H_2 molecule reached the bound state. The start time was the start of the simulation.
c) “Tunnel out” refers to the tunnel that the H_2 molecule used for unbinding.
d) “Time of the unbound state (ps)” refers to the time in the simulation when the unbound state was achieved.
e) “First Passage Time - Unbinding (ps)” is the time it took the H_2 molecule to reach the unbound state, starting from the bound state.
f) NA means ‘Not Available’. In some cases, especially near the end of the simulation, only binding events were captured and no unbinding events could be achieved.

Table S2. H_2 binding and unbinding events captured in 75 independent UMD simulations (250 ns length) of Mdg hydrogenase.

Replica #	Tunnels in[a]	First Passage Time - Binding[b] (ps)	Tunnels out[c]	Time of the unbound state[d] (ps)	First Passage Time - Unbinding[e] (ps)
4	T1	20570	T2	21450	880
6	T2	194680	T2	196670	1990
9	T3	31990	T1	32280	290
11	T2	5580	T2	10610	5030
12	T2	14650	T2	14860	210
12	T1	121400	T2	127380	5980
15	T2	108700	T5	123450	14750
16	T8	53310	T3	53710	400
17	T2	225720	T2	227210	1490
18	T3	106130	T2	106930	800
18	T8	106800	T2	111970	5170
20	T2	133290	T2	135220	1930
20	T2	151610	T2	155360	3750
20	T2	94780	T2	97080	2300
20	T2	235820	T2	238300	2480
22	T8	29090	T3	39300	10210
22	T2	65010	T3	72280	7270
22	T8	230090	T5	250000	19910
23	T2	235270	T2	236920	1650
25	T1	49750	T2	51340	1590
27	T1	2580	T2	5750	3170
27	T2	36460	T2	36790	330
27	T1	32090	T2	33380	1290
27	T2	67150	T1	68670	1520
27	T2	139340	T2	140200	860
27	T2	92130	T1	92860	730
27	T2	218060	T2	219880	1820
27	T2	29040	T2	30080	1040
27	T2	3490	T2	4020	530
27	T2	140340	T2	142570	2230
27	T2	3770	T2	4460	690
28	T1	53160	T2	58270	5110
28	T8	132340	T1	133950	1610
28	T2	97490	T8	98660	1170
28	T2	138490	T2	139600	1110
31	T2	45500	T1	47810	2310

31	T2	223130	T1	229020	5890
35	T2	77170	T2	81370	4200
36	T2	19050	T2	23540	4490
36	T3	11870	T1	12980	1110
36	T3	41360	T1	42890	1530
36	T8	13260	T8	18430	5170
36	T8	24740	T2	27710	2970
36	T2	18150	T2	21280	3130
36	T3	17500	T2	18390	890
36	T1	174410	T1	176190	1780
36	T3	18620	T2	19830	1210
36	T1	14460	T2	15060	600
39	T2	85900	T1	87140	1240
39	T8	32330	T8	33440	1110
39	T1	105280	T8	111700	6420
39	T2	95420	T2	97630	2210
39	T2	123740	T2	124120	380
39	T2	91090	T5	92920	1830
39	T1	18980	T1	20240	1260
40	T8	48510	T2	49930	1420
40	T1	78750	T2	79640	890
41	T4	169470	NA[f]	NA[f]	NA[f]
43	T2	27590	T1	28150	560
44	T3	185790	T8	186350	560
44	T1	45740	T1	46960	1220
44	T2	69250	T2	73040	3790
45	T2	119080	T3	120780	1700
45	T1	107110	T2	108670	1560
46	T2	111110	T2	112120	1010
46	T2	155610	T2	156120	510
47	T1	60020	T3	60270	250
47	T3	42370	T2	43040	670
47	T3	35950	T2	36770	820
47	T1	24100	T1	29750	5650
47	T2	72730	T1	75040	2310
47	T3	84400	T2	85090	690
47	T2	41820	T1	44540	2720
47	T1	47090	T2	47400	310
48	T8	45480	T3	46140	660
49	T1	111160	T2	115060	3900
50	T1	189260	T2	192720	3460

53	T2	48390	T2	58740	10350
61	T2	17260	T2	18130	870
61	T8	209810	T2	214080	4270
61	T1	16630	T8	22640	6010
61	T2	17030	T2	19060	2030
61	T3	13880	T3	15490	1610
61	T8	18370	T1	19710	1340
61	T3	16180	T1	16710	530
61	T2	20020	T2	22460	2440
63	T1	26500	T3	35430	8930
65	T4	198810	T4	214260	15450
65	T8	51370	T2	64750	13380
69	T1	68860	T2	69720	860
69	T3	80790	T2	82630	1840
69	T8	68550	T1	79630	11080
69	T1	36880	T1	38290	1410
70	T8	90320	T1	91140	820
71	T2	16720	T2	19300	2580
71	T2	13470	T1	16020	2550
74	T2	170310	T2	171200	890
74	T2	170520	T3	174100	3580
75	T2	89860	T1	92000	2140

a) "Tunnel in" refers to the tunnel that the H_2 molecule used for binding.
b) "First Passage Time - Binding (ps)" refers to the time in the simulation when the H_2 molecule reached the bound state. The start time was the start of the simulation.
c) "Tunnel out" refers to the tunnel that the H_2 molecule used for unbinding.
d) "Time of the unbound state (ps)" refers to the time in the simulation when the unbound state was achieved.
e) "First Passage Time - Unbinding (ps)" is the time it took the H_2 molecule to reach the unbound state, starting from the bound state.
f) NA means 'Not Available'. In some cases, especially near the end of the simulation, only binding events were captured and no unbinding events could be achieved.

Table S3. O_2 and CO binding and unbinding events captured in 75 and 150 independent UMD simulations (250 ns length), respectively, for Df hydrogenase.

Molecule	Replica #	Tunnels in[a]	First Passage Time - Binding[b] (ps)	Tunnels out[c]	Time of the unbound state[d] (ps)	First Passage Time - Unbinding[e] (ps)
O_2	29	T2	7460	T2	45530	38070
O_2	4	T1	236550	T1	246800	10250
O_2	50	T1	188890	T8	230350	41460
O_2	62	T2	62400	T1	72410	10010
CO	27	T1	161400	T1	211270	49870
CO	54	T1	196670	NA[f]	NA[f]	NA[f]

a) "Tunnel in" refers to the tunnel that the gas molecule used for binding.

b) "First Passage Time - Binding (ps)" refers to the time in the simulation when the gas molecule reached the bound state. The start time was the start of the simulation.

c) "Tunnel out" refers to the tunnel that the gas molecule used for unbinding.

d) "Time of the unbound state (ps)" refers to the time in the simulation when the unbound state was achieved.

e) "First Passage Time - Unbinding (ps)" is the time it took the gas molecule to reach the unbound state, starting from the bound state.

f) NA means 'Not Available'. In some cases, especially near the end of the simulation, only binding events were captured and no unbinding events could be achieved.

Table S4. Percentages (number of events) of paths used for binding and unbinding in Df and Mdg hydrogenases (raw data of Figure 3).

	T1	T2	T3	T4	T5	T6	T7	T8	T9
Df hydrogenase - Binding	25.5% (11)	46.5% (20)	11.6% (5)	11.6% (5)	0.0% (0)	0.0% (0)	0.0% (0)	4.6% (2)	0.0% (0)
Df hydrogenase - Unbinding	31.7% (13)	24.3% (10)	4.8% (2)	9.7% (4)	7.3% (3)	0.0% (0)	2.4% (1)	19.5% (8)	0.0% (0)
Mdg hydrogenase - Binding	22.2% (22)	47.4% (47)	13.1% (13)	2.0% (2)	0.0% (0)	0.0% (0)	0.0% (0)	15.1% (15)	0.0% (0)
Mdg hydrogenase - Unbinding	23.4% (23)	57.1% (56)	9.1% (9)	1.0% (1)	3.0% (3)	0.0% (0)	0.0% (0)	6.1% (6)	0.0% (0)

Table S5. Chi-square contributions per pathway in the comparison between UMD unbinding and UMD binding path probabilities for Df hydrogenase.

Pathway	UMD Binding	UMD Unbinding	Total contribution
T8	1.90	1.99	3.89
T5	1.54	1.61	3.15
T2	1.40	1.47	2.88
T3	0.56	0.59	1.15
T7	0.51	0.54	1.05
T1	0.13	0.14	0.28
T4	0.03	0.04	0.07
Sum			12.46
P-value			0.053

Table S6. Chi-square contributions per pathway in the comparison between UMD unbinding and UMD binding path probabilities for Mdg hydrogenase.

Pathway	UMD Binding	UMD Unbinding	Total contribution
T8	1.87	1.89	3.77
T5	1.51	1.52	3.03
T2	0.44	0.44	0.88
T3	0.34	0.35	0.69
T4	0.16	0.16	0.32
T1	0.02	0.02	0.03
Sum			8.72
P-value			0.121

Table S7. Chi-square contributions per pathway in the comparison between UMD unbinding and τRAMD unbinding path probabilities for Df hydrogenase.

Pathway	τRAMD Unbinding	UMD Unbinding	Total contribution
T2	4.24	7.65	11.89
T7	0.64	1.16	1.80
T1	0.63	1.14	1.78
T9	0.59	1.07	1.66
T4	0.28	0.51	0.79
T8	0.22	0.39	0.61
T6	0.20	0.36	0.55
T5	0.05	0.09	0.14
T3	0.01	0.03	0.04
Sum			19.27
P-value			0.013

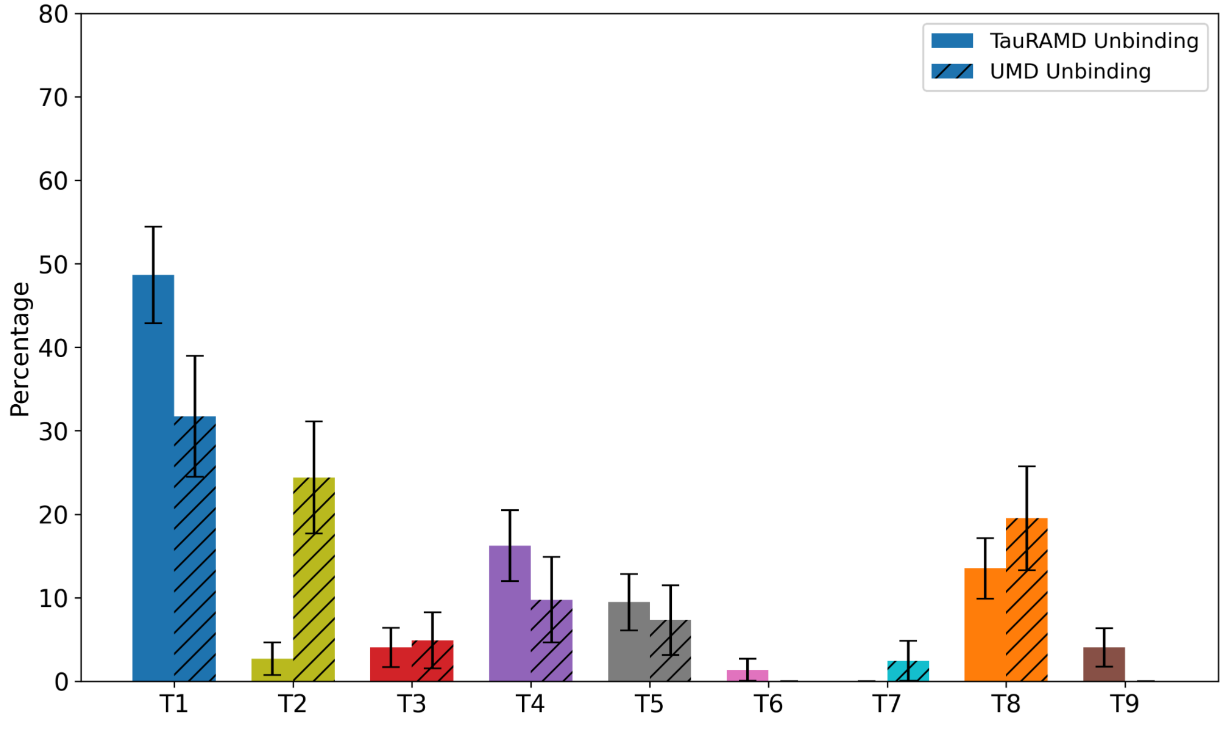

Figure S1. Comparison of pathway probabilities for unbinding events obtained from UMD and τRAMD simulations in Df hydrogenase.

Table S8. Chi-square contributions per pathway in the comparison between UMD unbinding and τRAMD unbinding path probabilities for Mdg hydrogenase.

Pathway	τRAMD Unbinding	UMD Unbinding	Total contribution
T2	15.11	11.56	26.67
T5	15.06	11.52	26.58
T6	8.14	6.23	14.37
T8	2.60	1.99	4.59
T3	2.57	1.96	4.53
T10	2.22	1.70	3.92
T4	2.21	1.69	3.91
T7	1.48	1.13	2.61
T1	0.03	0.03	0.06
Sum			87.24
P-value			$1.7e^{-15}$

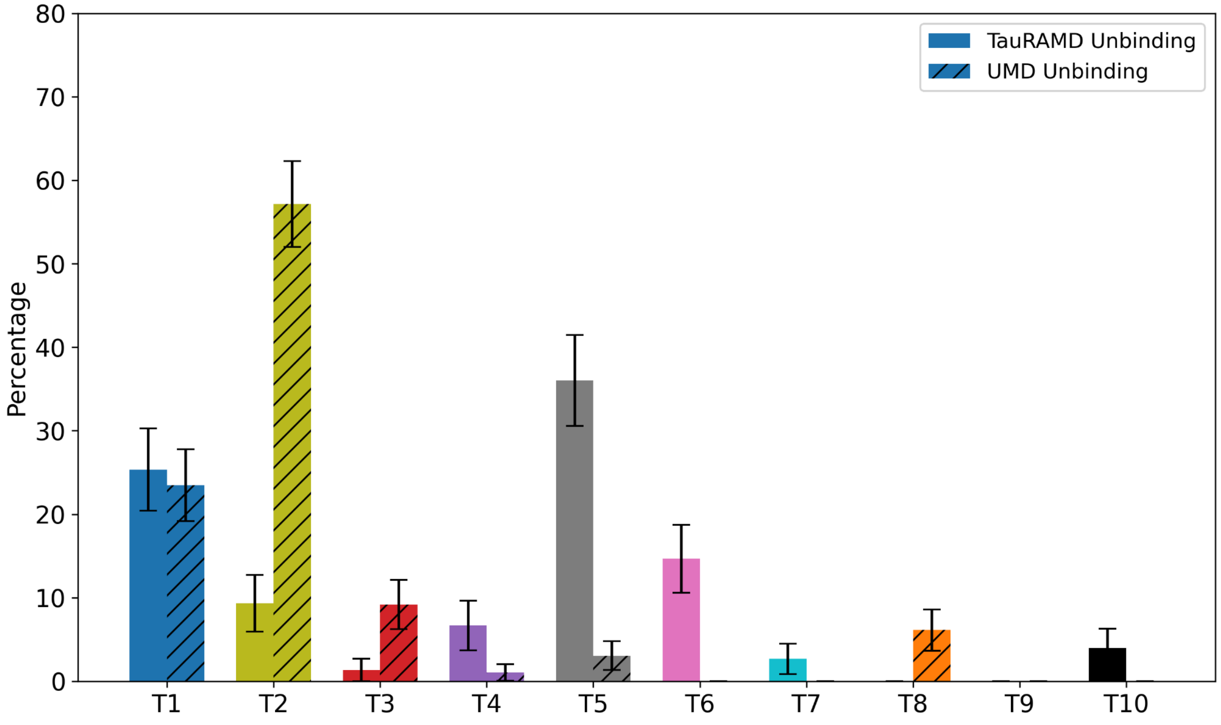

Figure S2. Comparison of pathway probabilities for unbinding events obtained from UMD and τRAMD simulations in Mdg hydrogenase.

Markov state model construction and validation for UMD simulations of Df hydrogenase

In order to find and characterize the states of the main bottleneck and build free energy landscapes, we constructed MSMs for Df hydrogenase and Mdg hydrogenase. This section describes the methods and results obtained for Df hydrogenase. The MSM was built using pyemma 2.5.2 program[31] and, as features, we used the pairwise distances between all heavy atoms of the V74 and L122 residues during the entire UMD simulations, which consist of 75 concatenated independent simulations of 250 ns each (37.5 μs in total). The high dimensional data was reduced using TICA with a lag time of 1 ns and 4 dimensions. Then, the kmeans method was used to cluster the configurations into 200 microstates. After the clustering, we obtained the discretized trajectories and the Markovian behavior was examined via the implied timescales plot (Figure S3). As shown in the figure, the plateau at longer timescales indicates Markovian behavior. After that we performed dimensionality reduction, clustering and obtained some discretized trajectories, and a MSM model with a lag time of 4 ns was constructed. Then, the Chapman-Kolmogorov test was used to validate the MSM model (Figure S4). Next, based on the implied timescales plot, the microstates were lumped into 4 main macrostates using the PCCA method[34] (Figure S5; the black and red dots represent the open and closed states of the bottleneck, respectively). The populations obtained for macrostates 1 (open state), 2 (closed state), 3 and 4 were 63.8%, 30.6%, 4.4% and 1.2%, respectively (Figure S6). The populations were calculated by summing the stationary probabilities of microstates in each macrostate. In the main text, we focused on the most populated states, open and closed states. Then, the rate matrix was generated in order to describe continuous-time transition rates between macrostates using the equation below:

$$K = \frac{1}{\tau}(P - I) \qquad (6)$$

In which **P** is the discrete-time transition probability matrix, **I** is the identity matrix, **τ** is the MSM lag time and **K** gives the rate from one state to another. Moreover, for the MSM macrostates, the MFPT can be calculated using equation 7. The MFPT can be interpreted as the

waiting time needed to go from one state to the other. Figures S7 and S8 show the macrostate transition network with the corresponding waiting times.

$$MFPT_{a \to b} \approx \frac{1}{k_{a \to b}} \tag{7}$$

A jupyter notebook containing all the code for the MSM model building is available at (https://github.com/FarzinSohraby/SI-H2ase-UMD).

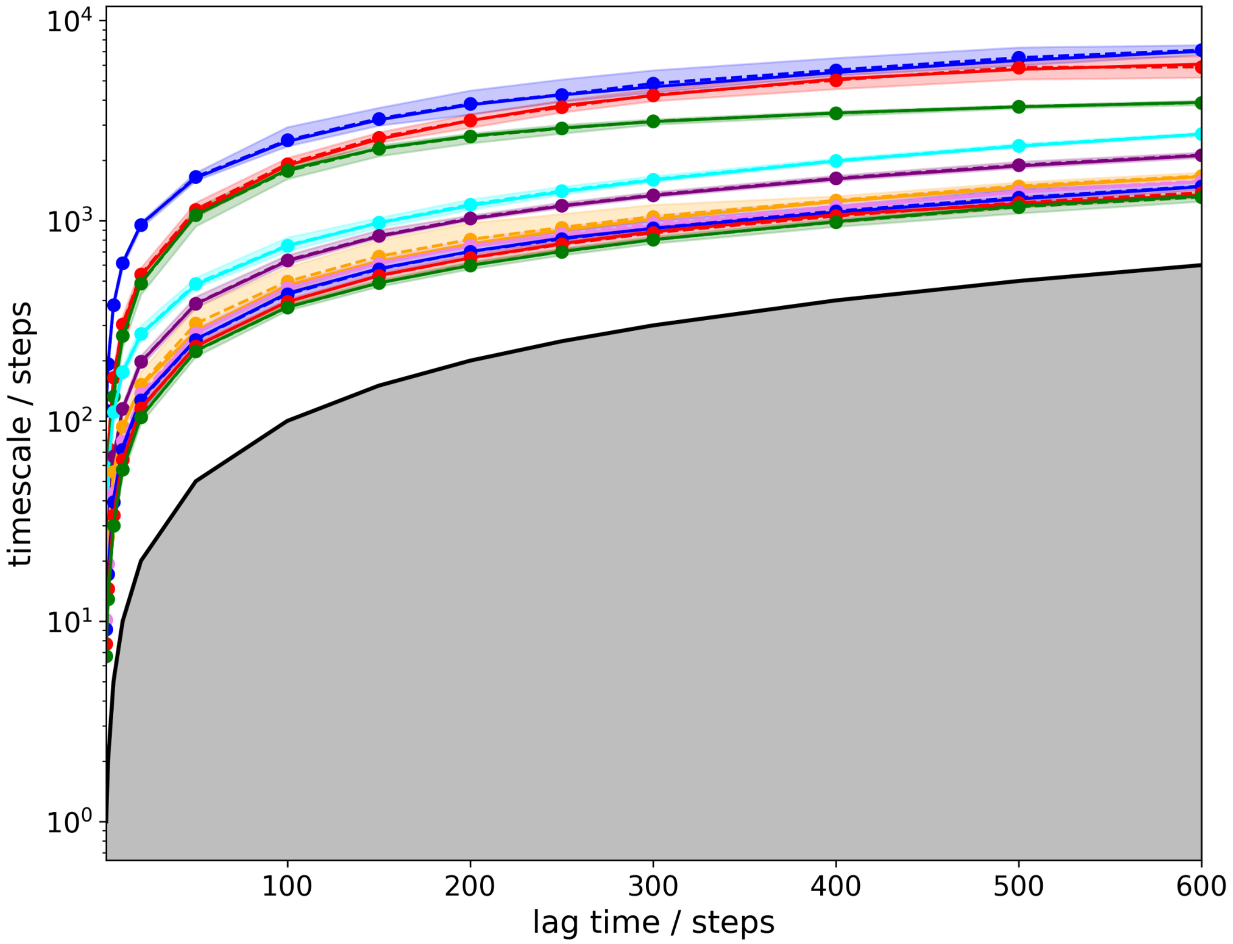

Figure S3. Implied timescales (ITS) analysis of the discretized trajectories using a Bayesian error estimate. Each colored line represents one of the top 10 slowest dynamical processes (ITS) extracted from the transition matrix at each lag time. Different colors distinguish between different timescales (eigenvalues of the MSM transition matrix). Solid lines represent the median estimated ITS for each lag time. Shaded areas (or if applicable, error bars/traced lines) reflect the Bayesian uncertainty (posterior confidence intervals) over the implied timescale estimates from bootstrapped MSM samples. The gray shaded region below the black curve indicates where the

estimated timescales fall below the lag time.

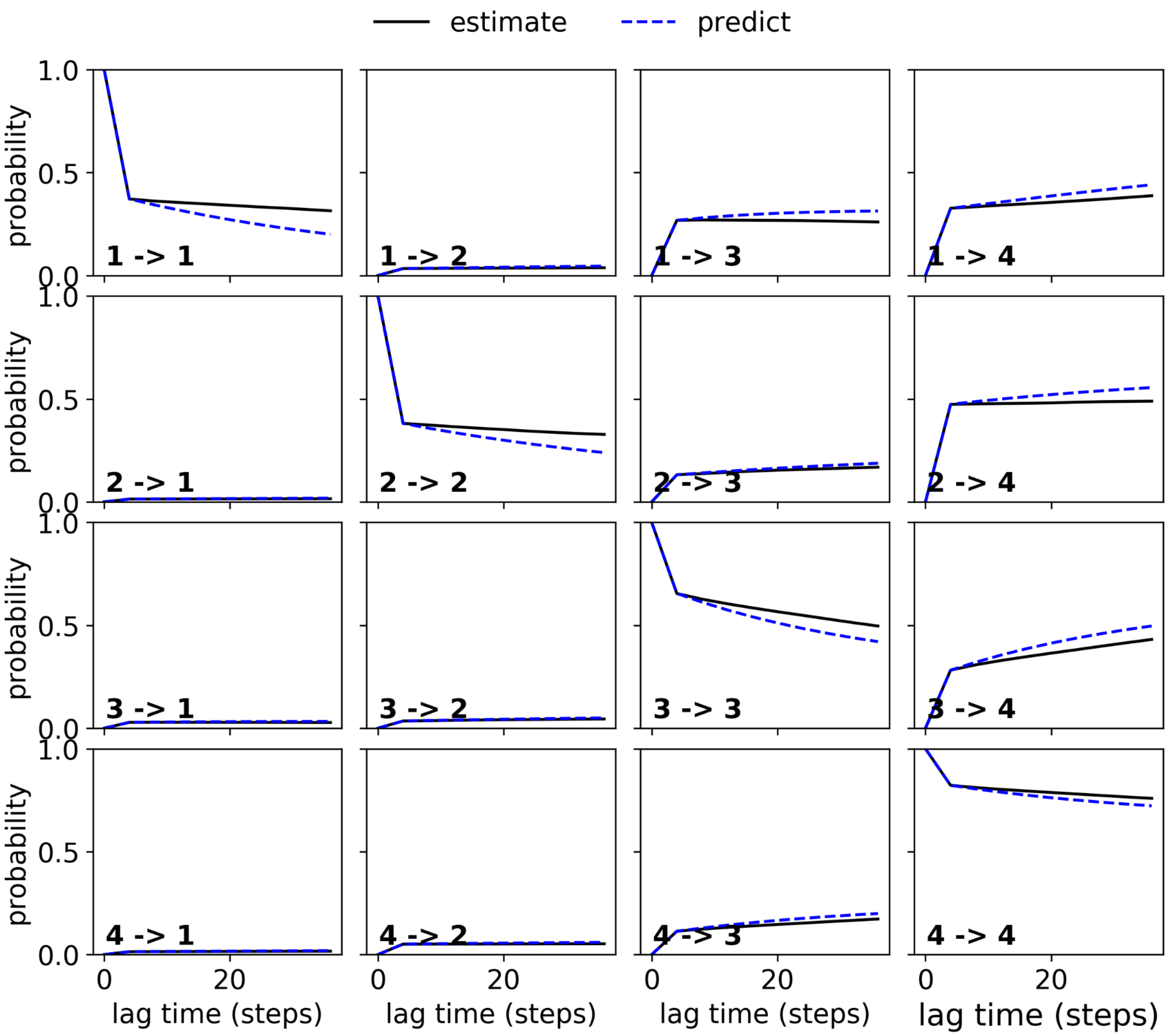

Figure S4. Chapman-Kolmogorov test for the constructed MSM model using 4 macrostates.

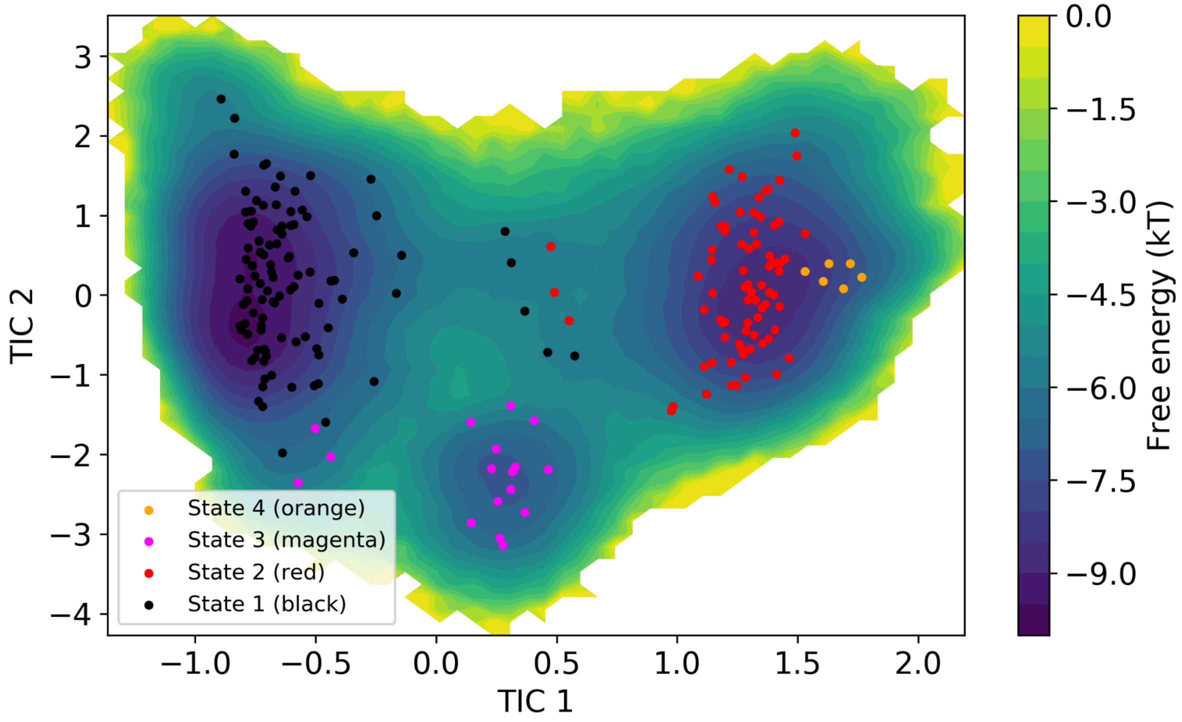

Figure S5. Free energy landscape with positions of microstates and macrostates obtained from MSM. Red and black dots represent the closed and open states. Each dot represents a microstate and is colored by its assigned macrostate.

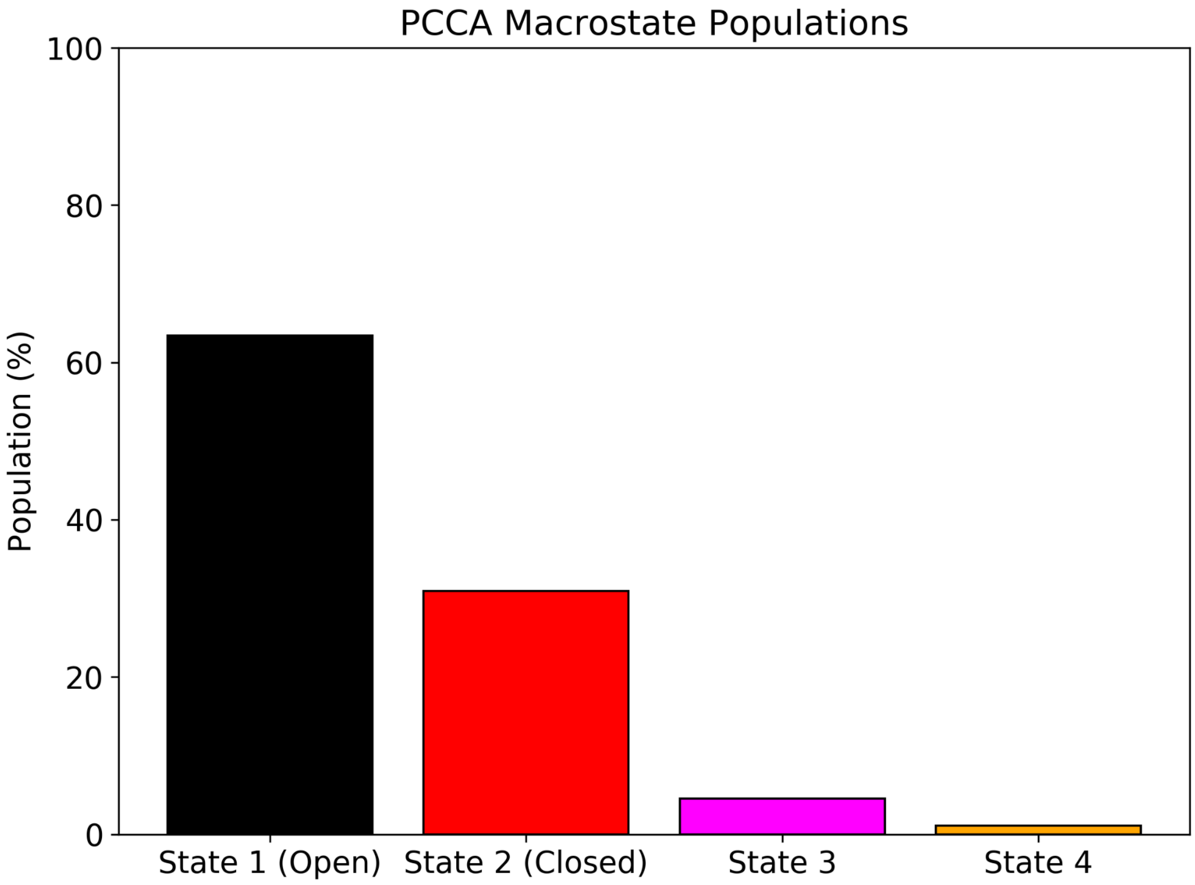

Figure S6. Populations of the 4 macrostates identified in MSM.

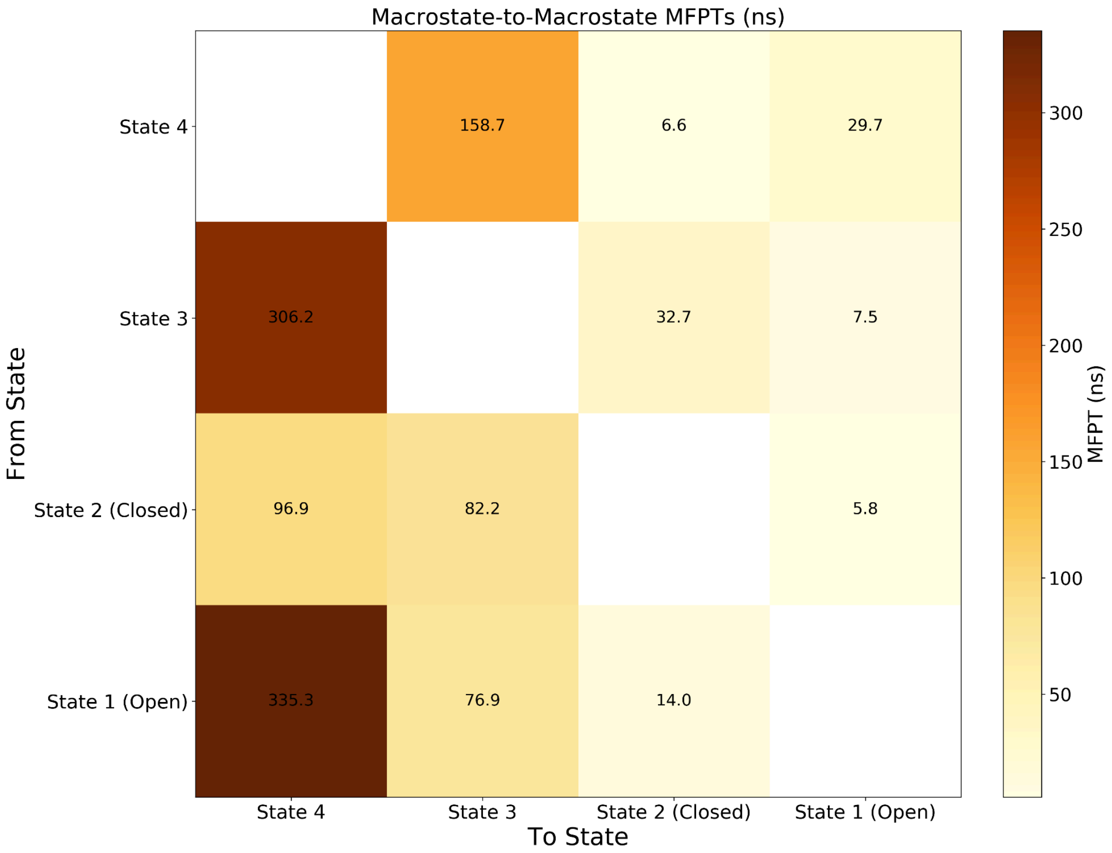

Figure S7. Matrix of the mean first passage times (MFPTs, in ns) between the four macrostates obtained from MSM.

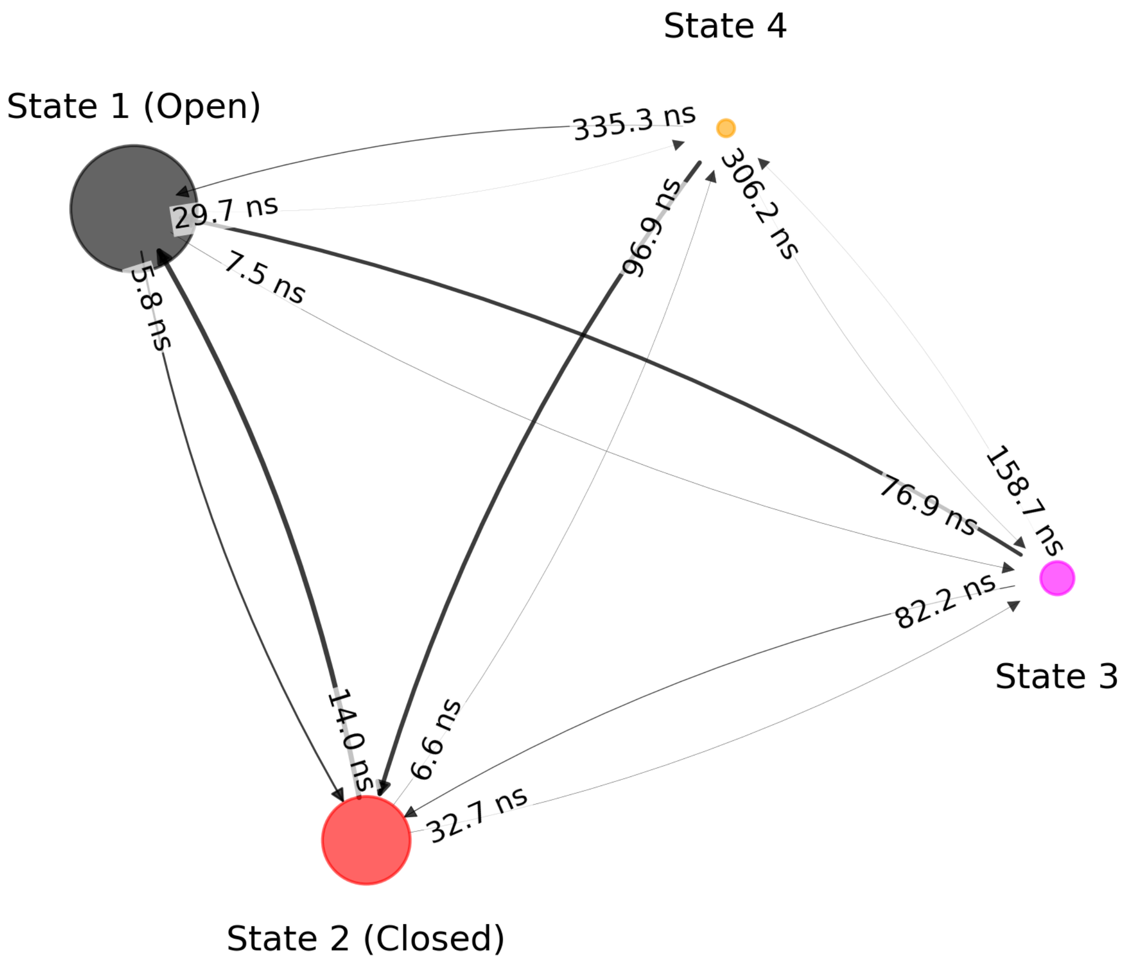

Figure S8. Transition network of the 4 macrostates obtained from MSM. The sizes of the nodes of the macrostates represent their population in the MSM.

DSSP analysis of the region around the L122 residue in Df hydrogenase

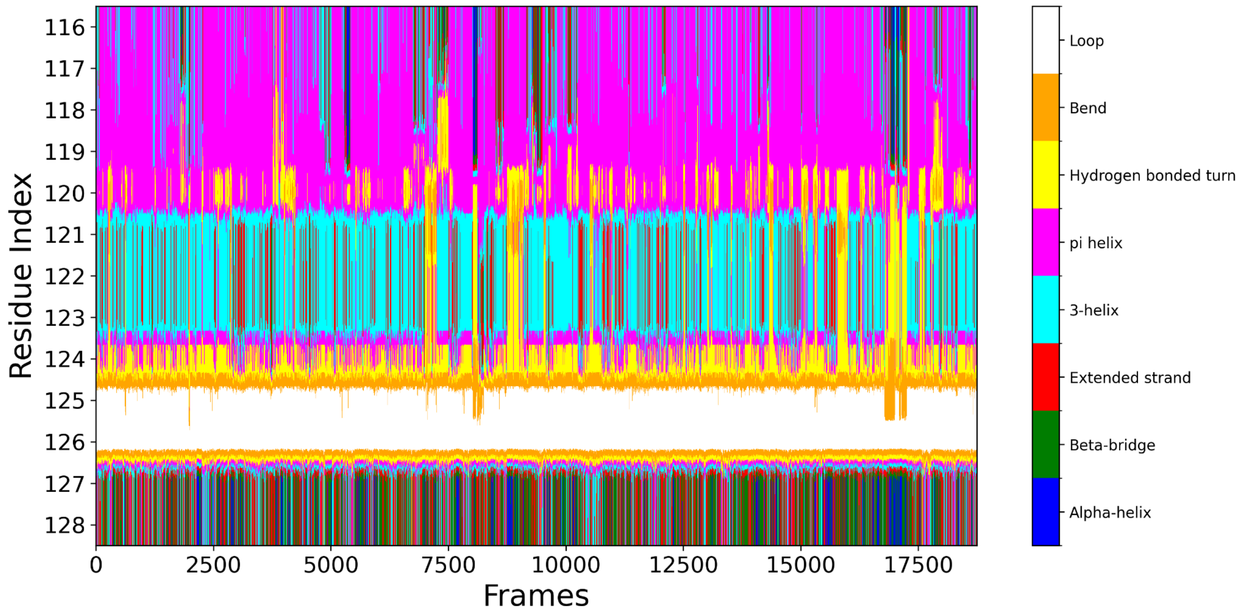

Figure S9. The DSSP analysis shows the changes in the secondary structure in the region around L122 in the UMD simulations for Df hydrogenase. The entire length of the concatenated UMD simulations was 18.75 μs (75 replicas), and frames were collected every 1 ns for the DSSP analysis, resulting in 18750 frames.

Table S9. Breck's kinetic diameter of small gas molecules, H_2, CO and O_2[33].

**Molecule**	**Diameter (Å)**
H_2	2.9
O_2	3.5
CO	3.8

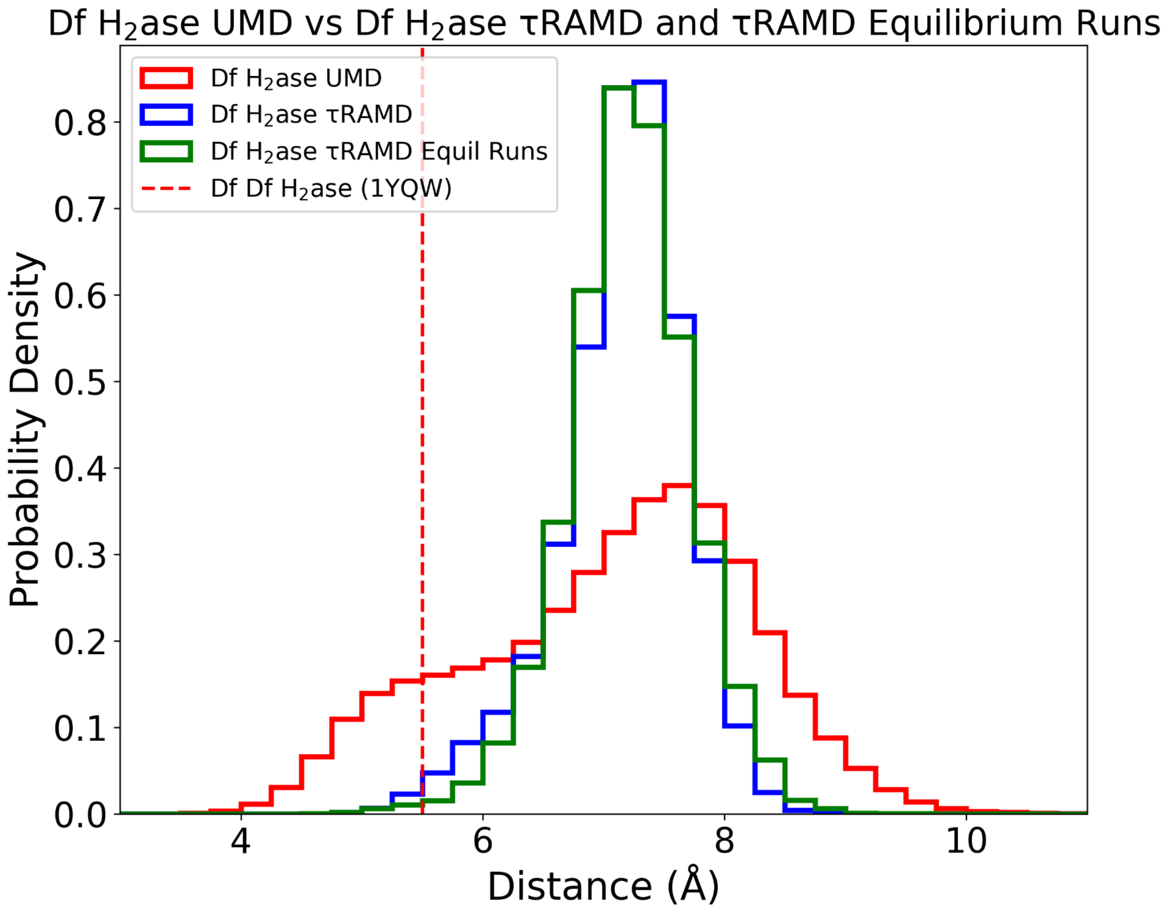

Figure S10. Probability density of lowest distance values between the bottleneck residues, V74 and L122, in UMD simulations of Df hydrogenase (75 replicas of 250 ns for H_2 binding and unbinding), in τRAMD simulations of Df hydrogenase (75 short replicas for H_2 unbinding) and in the equilibrium simulations performed prior to the τRAMD simulations (one trajectory of 50 ns, 5 trajectories of 20 ns, unbiased MD simulations with positional restraints to keep H_2 in the bound state). The distance value in the crystallographic structure is shown as a traced line.

Structural analysis of the bottleneck in Mdg hydrogenase

We investigated the motions of the V74-L122 bottleneck in Mdg hydrogenase to test if the open and closed states found in Df hydrogenase were also found in other hydrogenases. The dynamics of V74 and L122 in the bottleneck of Mdg hydrogenase was minor compared to the bottleneck in the Df hydrogenase. The MSM-reweighted free energy landscape showed the presence of two states (Figure S11A). However, the structural differences between these two

states were smaller in comparison to the ones observed in Df hydrogenase (Figure S11B), and we do not observe a clear open state as in the bottleneck of Df hydrogenase. The DSSP analysis shows that there are no significant changes in the secondary structure of L122 and its surrounding residues (Figure S11C).

The reweighted free energy landscape was produced using the MSM built for the MD simulations of Mdg hydrogenase. TICA with a lag time of 1 ns was used to reduce the high dimensional data and then the configurations were reduced into 200 microstates using the kmeans method. A MSM with a lag time of 2 ns was built.

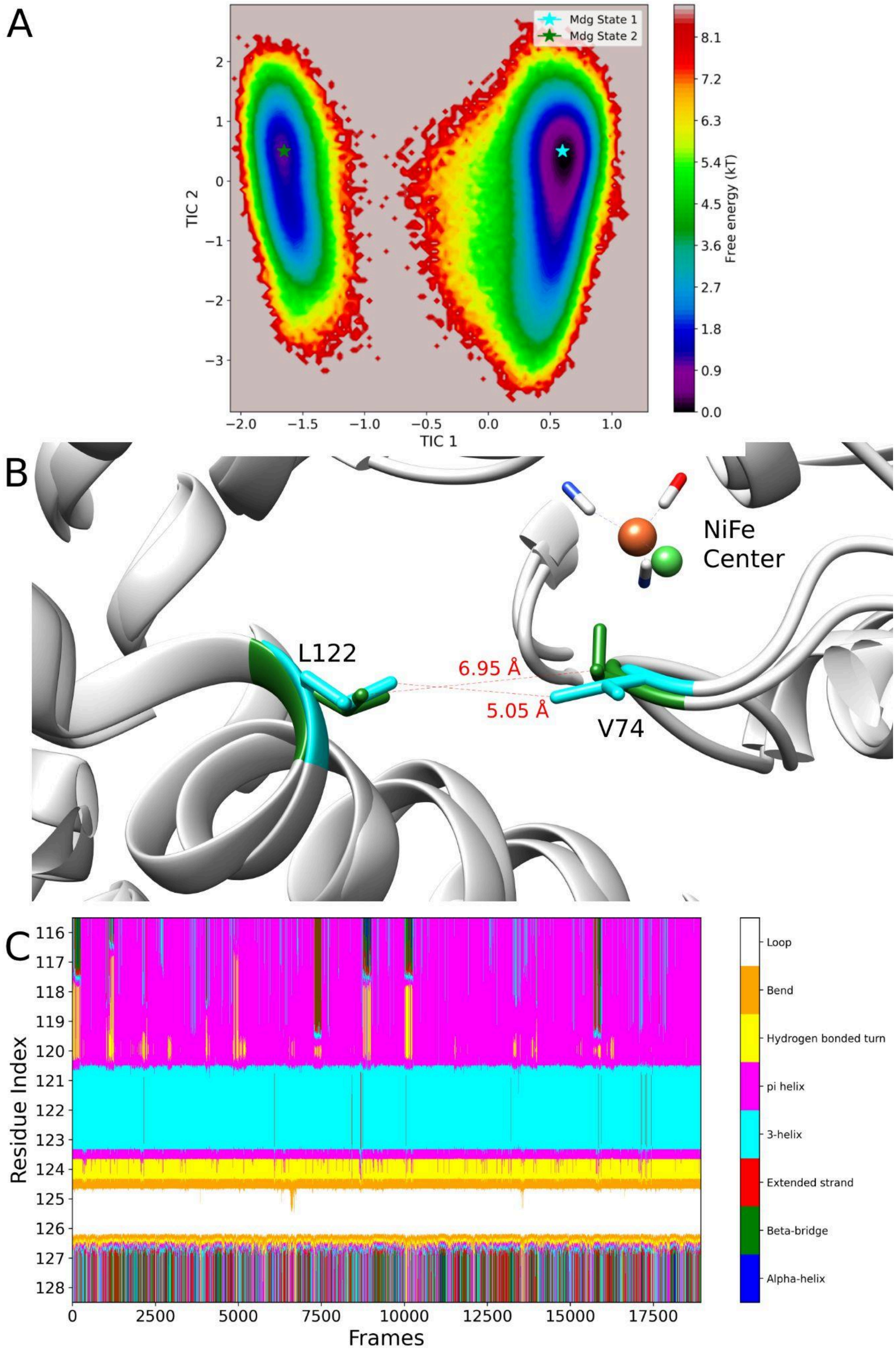

Figure S11. Structural analysis of the Mdg hydrogenase bottleneck. A) Free energy landscape of the V74-L122 bottleneck in Mdg hydrogenase computed using a Markov state model. Two states

were identified, states 1 and 2. The stars mark the location of the snapshots of the simulation shown in panel B. B) Representative snapshots of states 1 and 2 of Mdg hydrogenase. C) DSSP analysis of the secondary structure of the region around residue L122. The entire length of the concatenated UMD simulations was 18.75 µs (75 replicas), and frames were collected every 1 ns for the DSSP analysis, resulting in 18750 frames.

References

(1) Volbeda, A.; Martin, L.; Cavazza, C.; Matho, M.; Faber, B. W.; Roseboom, W.; Albracht, S. P. J.; Garcin, E.; Rousset, M.; Fontecilla-Camps, J. C. Structural Differences between the Ready and Unready Oxidized States of [NiFe] Hydrogenases. *JBIC J. Biol. Inorg. Chem.* **2005**, *10* (3), 239–249. https://doi.org/10.1007/s00775-005-0632-x.

(2) Berman, H. M. The Protein Data Bank. *Nucleic Acids Res.* **2000**, *28* (1), 235–242. https://doi.org/10.1093/nar/28.1.235.

(3) Smith, D. M. A.; Xiong, Y.; Straatsma, T. P.; Rosso, K. M.; Squier, T. C. Force-Field Development and Molecular Dynamics of [NiFe] Hydrogenase. *J. Chem. Theory Comput.* **2012**, *8* (6), 2103–2114. https://doi.org/10.1021/ct300185u.

(4) Teixeira, V. H.; Baptista, A. M.; Soares, C. M. Pathways of H2 toward the Active Site of [NiFe]-Hydrogenase. *Biophys. J.* **2006**, *91* (6), 2035–2045. https://doi.org/10.1529/biophysj.106.084376.

(5) Liebgott, P.-P.; Leroux, F.; Burlat, B.; Dementin, S.; Baffert, C.; Lautier, T.; Fourmond, V.; Ceccaldi, P.; Cavazza, C.; Meynial-Salles, I.; Soucaille, P.; Fontecilla-Camps, J. C.; Guigliarelli, B.; Bertrand, P.; Rousset, M.; Léger, C. Relating Diffusion along the Substrate Tunnel and Oxygen Sensitivity in Hydrogenase. *Nat. Chem. Biol.* **2010**, *6* (1), 63–70. https://doi.org/10.1038/nchembio.276.

(6) Li, H.; Robertson, A. D.; Jensen, J. H. Very Fast Empirical Prediction and Rationalization of Protein pKa Values. *Proteins Struct. Funct. Bioinforma.* **2005**, *61* (4), 704–721. https://doi.org/10.1002/prot.20660.

(7) Bas, D. C.; Rogers, D. M.; Jensen, J. H. Very Fast Prediction and Rationalization of pKa Values for Protein-Ligand Complexes. *Proteins Struct. Funct. Bioinforma.* **2008**, *73* (3), 765–783. https://doi.org/10.1002/prot.22102.

(8) Olsson, M. H. M.; Søndergaard, C. R.; Rostkowski, M.; Jensen, J. H. PROPKA3: Consistent Treatment of Internal and Surface Residues in Empirical p K_a Predictions. *J. Chem. Theory Comput.* **2011**, *7* (2), 525–537. https://doi.org/10.1021/ct100578z.

(9) Unni, S.; Huang, Y.; Hanson, R. M.; Tobias, M.; Krishnan, S.; Li, W. W.; Nielsen, J. E.; Baker, N. A. Web Servers and Services for Electrostatics Calculations with APBS and PDB2PQR. *J. Comput. Chem.* **2011**, *32* (7), 1488–1491. https://doi.org/10.1002/jcc.21720.

(10) Dolinsky, T. J.; Nielsen, J. E.; McCammon, J. A.; Baker, N. A. PDB2PQR: An Automated Pipeline for the Setup of Poisson-Boltzmann Electrostatics Calculations. *Nucleic Acids Res.* **2004**, *32* (Web Server), W665–W667. https://doi.org/10.1093/nar/gkh381.

(11) Wang, S.; Hou, K.; Heinz, H. Accurate and Compatible Force Fields for Molecular Oxygen, Nitrogen, and Hydrogen to Simulate Gases, Electrolytes, and Heterogeneous Interfaces. *J. Chem. Theory Comput.* **2021**, *17* (8), 5198–5213. https://doi.org/10.1021/acs.jctc.0c01132.

(12) Mark, P.; Nilsson, L. Structure and Dynamics of the TIP3P, SPC, and SPC/E Water Models at 298 K. *J. Phys. Chem. A* **2001**, *105* (43), 9954–9960. https://doi.org/10.1021/jp003020w.

(13) Abraham, M. J.; Murtola, T.; Schulz, R.; Páll, S.; Smith, J. C.; Hess, B.; Lindahl, E. GROMACS: High Performance Molecular Simulations through Multi-Level Parallelism from Laptops to Supercomputers. *SoftwareX* **2015**, *1–2*, 19–25. https://doi.org/10.1016/j.softx.2015.06.001.

(14) Ponder, J. W.; Case, D. A. Force Fields for Protein Simulations. In *Advances in Protein Chemistry*; Elsevier, 2003; Vol. 66, pp 27–85. https://doi.org/10.1016/S0065-3233(03)66002-X.
(15) Sohraby, F.; Nunes-Alves, A. Characterization of the Bottlenecks and Pathways for Inhibitor Dissociation from [NiFe] Hydrogenase. *J. Chem. Inf. Model.* **2024**, *64* (10), 4193–4203. https://doi.org/10.1021/acs.jcim.4c00187.
(16) Sohraby, F.; Guo, J.-Y.; Nunes-Alves, A. PathInHydro, a Set of Machine Learning Models to Identify Unbinding Pathways of Gas Molecules in [NiFe] Hydrogenases. *J. Chem. Inf. Model.* **2025**, *65* (2), 589–602. https://doi.org/10.1021/acs.jcim.4c01656.
(17) Berendsen, H. J. C.; Postma, J. P. M.; Van Gunsteren, W. F.; DiNola, A.; Haak, J. R. Molecular Dynamics with Coupling to an External Bath. *J. Chem. Phys.* **1984**, *81* (8), 3684–3690. https://doi.org/10.1063/1.448118.
(18) Nosé, S. A Molecular Dynamics Method for Simulations in the Canonical Ensemble. *Mol. Phys.* **1984**, *52* (2), 255–268. https://doi.org/10.1080/00268978400101201.
(19) Hoover, W. G. Canonical Dynamics: Equilibrium Phase-Space Distributions. *Phys. Rev. A* **1985**, *31* (3), 1695–1697. https://doi.org/10.1103/PhysRevA.31.1695.
(20) Parrinello, M.; Rahman, A. Polymorphic Transitions in Single Crystals: A New Molecular Dynamics Method. *J. Appl. Phys.* **1981**, *52* (12), 7182–7190. https://doi.org/10.1063/1.328693.
(21) Nosé, S.; Klein, M. L. Constant Pressure Molecular Dynamics for Molecular Systems. *Mol. Phys.* **1983**, *50* (5), 1055–1076. https://doi.org/10.1080/00268978300102851.
(22) Hess, B.; Bekker, H.; Berendsen, H. J. C.; Fraaije, J. G. E. M. LINCS: A Linear Constraint Solver for Molecular Simulations. *J. Comput. Chem.* **1997**, *18* (12), 1463–1472. https://doi.org/10.1002/(SICI)1096-987X(199709)18:12<1463::AID-JCC4>3.0.CO;2-H.
(23) Yakovlev, D.; Boek, E. S. Structure of Bilayer Membranes of Gemini Surfactants with Rigid and Flexible Spacers from MD Simulations. In *Computational Science — ICCS 2003*; Sloot, P. M. A., Abramson, D., Bogdanov, A. V., Gorbachev, Y. E., Dongarra, J. J., Zomaya, A. Y., Eds.; Goos, G., Hartmanis, J., Van Leeuwen, J., Series Eds.; Lecture Notes in Computer Science; Springer Berlin Heidelberg: Berlin, Heidelberg, 2003; Vol. 2658, pp 668–677. https://doi.org/10.1007/3-540-44862-4_72.
(24) Darden, T.; York, D.; Pedersen, L. Particle Mesh Ewald: An $N \cdot \log(N)$ Method for Ewald Sums in Large Systems. *J. Chem. Phys.* **1993**, *98* (12), 10089–10092. https://doi.org/10.1063/1.464397.
(25) Cheatham, T. E. I.; Miller, J. L.; Fox, T.; Darden, T. A.; Kollman, P. A. Molecular Dynamics Simulations on Solvated Biomolecular Systems: The Particle Mesh Ewald Method Leads to Stable Trajectories of DNA, RNA, and Proteins. *J. Am. Chem. Soc.* **1995**, *117* (14), 4193–4194. https://doi.org/10.1021/ja00119a045.
(26) Chovancova, E.; Pavelka, A.; Benes, P.; Strnad, O.; Brezovsky, J.; Kozlikova, B.; Gora, A.; Sustr, V.; Klvana, M.; Medek, P.; Biedermannova, L.; Sochor, J.; Damborsky, J. CAVER 3.0: A Tool for the Analysis of Transport Pathways in Dynamic Protein Structures. *PLoS Comput. Biol.* **2012**, *8* (10), e1002708. https://doi.org/10.1371/journal.pcbi.1002708.
(27) Gowers, R.; Linke, M.; Barnoud, J.; Reddy, T.; Melo, M.; Seyler, S.; Domański, J.; Dotson, D.; Buchoux, S.; Kenney, I.; Beckstein, O. MDAnalysis: A Python Package for the Rapid Analysis of Molecular Dynamics Simulations; Austin, Texas, 2016; pp 98–105. https://doi.org/10.25080/Majora-629e541a-00e.
(28) Michaud‐Agrawal, N.; Denning, E. J.; Woolf, T. B.; Beckstein, O. MDAnalysis: A Toolkit for the Analysis of Molecular Dynamics Simulations. *J. Comput. Chem.* **2011**, *32* (10), 2319–2327. https://doi.org/10.1002/jcc.21787.
(29) McGibbon, R. T.; Beauchamp, K. A.; Harrigan, M. P.; Klein, C.; Swails, J. M.; Hernández, C. X.; Schwantes, C. R.; Wang, L.-P.; Lane, T. J.; Pande, V. S. MDTraj: A Modern Open Library for the Analysis of Molecular Dynamics Trajectories. *Biophys. J.* **2015**, *109* (8),

1528–1532. https://doi.org/10.1016/j.bpj.2015.08.015.
(30) Pettersen, E. F.; Goddard, T. D.; Huang, C. C.; Couch, G. S.; Greenblatt, D. M.; Meng, E. C.; Ferrin, T. E. UCSF Chimera?A Visualization System for Exploratory Research and Analysis. *J. Comput. Chem.* **2004**, *25* (13), 1605–1612. https://doi.org/10.1002/jcc.20084.
(31) Scherer, M. K.; Trendelkamp-Schroer, B.; Paul, F.; Pérez-Hernández, G.; Hoffmann, M.; Plattner, N.; Wehmeyer, C.; Prinz, J.-H.; Noé, F. PyEMMA 2: A Software Package for Estimation, Validation, and Analysis of Markov Models. *J. Chem. Theory Comput.* **2015**, *11* (11), 5525–5542. https://doi.org/10.1021/acs.jctc.5b00743.
(32) Leroux, F.; Dementin, S.; Burlat, B.; Cournac, L.; Volbeda, A.; Champ, S.; Martin, L.; Guigliarelli, B.; Bertrand, P.; Fontecilla-Camps, J.; Rousset, M.; Léger, C. Experimental Approaches to Kinetics of Gas Diffusion in Hydrogenase. *Proc. Natl. Acad. Sci.* **2008**, *105* (32), 11188–11193. https://doi.org/10.1073/pnas.0803689105.
(33) Donald W. Breck. *Zeolite Molecular Sieves: Structure, Chemistry and Use*; John Wiley & Sons Inc, 1974.
(34) Schütte, C.; Fischer, A.; Huisinga, W.; Deuflhard, P. A Direct Approach to Conformational Dynamics Based on Hybrid Monte Carlo. *J. Comput. Phys.* **1999**, *151* (1), 146–168. https://doi.org/10.1006/jcph.1999.6231.
