# Supplementary material for: Symmetric Ligand Binding Pathways and Dual-State Bottleneck in [NiFe] Hydrogenases from Unbiased Molecular Dynamics
Source: J Phys Chem Lett. 2025 Jul 29;16(31):7960–7. doi: 10.1021/acs.jpclett.5c01673 (PMC12337149; doi:10.1021/acs.jpclett.5c01673)
Supplement: Supplementary file 2 [file jz5c01673_si_002.pdf]

jz-2025-01673u.R1

Name: Peer Review Information for "Symmetric Ligand Binding Pathways and Dual-State Bottleneck in [NiFe] Hydrogenases from Unbiased Molecular Dynamics"

First Round of Reviewer Comments

Reviewer: 1

Comments to the Author

This is an exciting work from Nunes lab that perform here unbiased MDs to describe kinetic and H2 (un)binding uathways for both open and closed forms of [NiFe] hydrogenases. This is a rather important for physical chemistry both as regards the minimal H2 ligand and unbiased MDs and should be published.

However, I would like from the authors to adrress the following to improve the presentation:

1) I have some experience with τRAMD (I have applied the algorithm to class A GPCRs) developed by Koch and Wade. In order to run this algorithm several repetition should be performed and the exit force must be carefully adjusted. τRAMD has been applied by the Nunes lab in their previous work with other systems and this protein system also. The τRAMD can work considering that RTs are compared with experimental values; these are calculated relative RTs. The authors while they are referred to enhanced sampling they should explain this.

2) What was the force applied to H2 to exit ? Did they find the same bottleneck here and with τRAMD? Did they ran τRAMD for both closed and open states?

3) Since here unbiased MDs which are computationally expensive and for the minimal H2 molecules were compared, then we may consider that for exit pathways τRAMD works for any system?

Technical part

4) Why the authors used for the protein ff99sb and not an earlier version? Even if they used it in earlier versions succesfully why they did not try a comparison eg with ff19sb? I do not ask to run that huge work again, nut at least add a comment either in main text or SI.

5) Why the authors performed their simulations at 40 oC?

6) Did they perform the simulations at gpus? This should be noted in the paper. How long as regards real time the MDs take to reach a completion?

Reviewer: 2

Comments to the Author

Major advance reported in the paper: The authors have used the process of H_2 binding to the hydrogenase enzyme to demonstrate how unbiased simulations can be utilized to study the (un)binding kinetics and pathways of fast binding ligands. They also show that the unbinding pathways sampled using biased sampling methods, such as tau-RAMD, agree well with the unbiased results.

Immediate significance of this advance: This is a significant discovery, as it demonstrates the feasibility of using computationally tractable short molecular simulations to study the kinetics of complex biomolecular systems of physiological relevance. There is a dire need for efficient computational methods to screen drug candidates based on unbinding kinetics, and this paper shows that short unbiased MD simulations can be used in that regard. However, the scope of this work is somewhat

limited by the fact that the authors only studied one simple fast-binding ligand, hydrogen, and did not show how their approach can be scaled to ligands with longer residence times.

Technical Suggestions: This paper can be published after addressing the following comments and concerns.

1. It is unclear why these two specific hydrogenases were chosen for this work, rather than a pharmaceutically relevant system like the FKBP protein, where the conclusions may be more transferable to other biological phenomena.
2. It should be mentioned in the text that many short simulations, totaling 40 microseconds, were performed instead of one long unbiased simulation. It should also be mentioned that equilibrium properties should not be estimated directly from this ensemble of disconnected simulations.
3. The authors mention observing both binding and unbinding events, but report only the k_{on} from their computational study. The k_{off} estimation from simulation, along with its agreement with the experimental values, should be provided in the main text.
4. Observing low binding/unbinding events with O_2 and CO may indicate that they bind strongly or their kinetics is slower. As the statistics are low, it may not be possible to be quantitative, but this qualitative effect should not be ignored, and their pathways should also be reported.
5. How the pathways of binding were classified as symmetric using the chi-square test is not clear. How is it possible to characterize a pathway using a single distance? How is the distribution of pathways computed?
6. Is the free energy landscape in Figure 4b computed using Markov state models (MSM) or some other method? Otherwise, how are multiple disconnected trajectories included together?
7. Minor style-related comment: The authors should just use the word “hydrogenase” instead of H_2ase. It does not reduce the word count and makes it difficult to read.

Author's Response to Peer Review Comments:

Point-by-point response to reviewers

We thank the reviewers for their constructive feedback and valuable suggestions on the manuscript. In the revised version, we have addressed all the points raised by the reviewers. We believe these revisions have further improved the quality of our work and the manuscript. The reviewer's comments have been separated into numbered comments and answers. The answers are in bold style and the text that has been added to the manuscript has been inserted here between quotation marks.
In addition, in the revised manuscript we improved the definition of the bound state.

"The bound state was achieved when H_2 reached a distance of 5 Å or lower to the center of mass of the [NiFe] center, and it was also near the interface of the Ni and Fe atoms of the [NiFe] center."

Reviewer(s)' Comments to Author:

Reviewer: 1

Recommendation: This paper is probably publishable, but major revision is needed; I do not need to see future revisions.

Comments: This is an exciting work from Nunes lab that perform here unbiased MDs to describe kinetic and H2 (un)binding uathways for both open and closed forms of [NiFe] hydrogenases. This is a rather important for physical chemistry both as regards the minimal H2 ligand and unbiased MDs and should be published.

However, I would like from the authors to adrress the following to improve the presentation:

Comment 1:
I have some experience with τRAMD (I have applied the algorithm to class A GPCRs) developed by Koch and Wade. In order to run this algorithm several repetition should be performed and the exit force must be carefully adjusted. τRAMD has been applied by the Nunes lab in their previous work with other systems and this protein system also. The τRAMD can work considering that RTs are compared with experimental values; these are calculated relative RTs. The authors while they are referred to enhanced sampling they should explain this.

Answer 1:

Following the reviewer's suggestion, we added a few sentences to the text for clarifying how τRAMD works.

"In previous works[13,35], we used τRAMD to simulate unbinding of H_2, CO and O_2 from Df and Mdg hydrogenases. τRAMD is an enhanced sampling technique which applies a force of constant magnitude and random orientation on the center of mass of the ligand to facilitate ligand unbinding. The relative residence times obtained with τRAMD, from tens of unbinding events for one protein-ligand complex, can be compared with experimental values for benchmarking, and can be used to rank multiple ligands or different mutants of a protein. We performed τRAMD simulations for 10 different mutants of Df hydrogenase, using a force with a magnitude of 1 kcal/(mol.Å) and 75 unbinding events to estimate relative residence times, and we could successfully reproduce the ranking of absolute residence times measured experimentally (R = 0.79, ρ = 0.75)."

Comment 2:

What was the force applied to H2 to exit ? Did they find the same bottleneck here and with τRAMD? Did they ran τRAMD for both closed and open states?

Answer 2:

The force magnitude applied to H_2 was 1 kcal/(mol.Å), since the gas molecules are smaller and have relatively less interactions and high diffusion rates in comparison to drug-like molecules. A paragraph has been added to the text for clarification about the details of the τRAMD runs in our previous works.

"We performed τRAMD simulations for 10 different mutants of Df hydrogenase, using a force with a magnitude of 1 kcal/(mol.Å) and 75 unbinding events to estimate relative residence times, and we could successfully reproduce the ranking of absolute residence times measured experimentally (R = 0.79, ρ = 0.75)."

Regarding the bottleneck, in the literature there were 2 hypotheses for the pair of residues which constitutes the main bottleneck that regulates the transit of gas molecules, H_2, O_2 and CO, to the catalytic site of the Df hydrogenase. One hypothesis was that the main bottleneck is between residues V74 and L122[5] residues but later it was reported that there is another bottleneck between R476 and V74[3,4]. In our previous work with τRAMD, we compared the experimental residence times of 10 mutants plus the WT form of Df hydrogenase with the average width of the 2 possible bottlenecks, obtained from tauRAMD simulations. We found a strong and negative correlation (R = -0.64) between the experimental residence times and the width of the V74 and L122 bottleneck. This shows that long residence times are associated with short bottleneck widths, and is a strong indication that the distance between residues 74 and 122 is the main bottleneck and controls the residence times in the Df hydrogenase. However, we found a lower positive correlation (R = 0.58) between the

experimental residence times and the width of the R476 and V74 bottleneck. The positive correlation shows that long residence times are associated with large bottleneck widths, which is an indication that the distance between residues R476 and V74 does not act as a bottleneck in the Df hydrogenase.
In the present manuscript, we investigated only the WT form of Df hydrogenase using UMD simulations. Therefore, it is not possible to calculate correlations between the residence times and the widths of candidate bottlenecks to identify bottlenecks for gas transit, as we did in previous work.
A sentence was added in the manuscript to explain how the bottleneck was identified:
"Additionally, we performed data analysis to investigate the dynamics of the main bottleneck for ligand unbinding, located between two evolutionary conserved hydrophobic residues, V74 and L122 (Df hydrogenase sequence numbering), in the UMD simulations. The bottleneck distance was identified by us[13] and others[37,39] as one of the main factors modulating the residence times for CO bound to different mutants of Df hydrogenaseH_2ase. In our previous work[13], we identified a strong correlation between the distances between residues V74 and L122 and the experimentally measured residence times, suggesting that these two residues act as a bottleneck for gas transit to the catalytic site."

During the τRAMD simulations, we only observed the open state of the bottleneck, possibly because the simulation times for equilibration prior τRAMD and for τRAMD were short. Despite running 75 replicas to simulate unbinding events of H_2 from Df hydrogenase, we were not able to capture the closed state of the bottleneck in the τRAMD simulations. We added one sentence in the main text and a figure in the supporting information to mention this observation.
"In the τRAMD simulations from previous work[13], we only observed the open state of the bottleneck (Figure S10)."

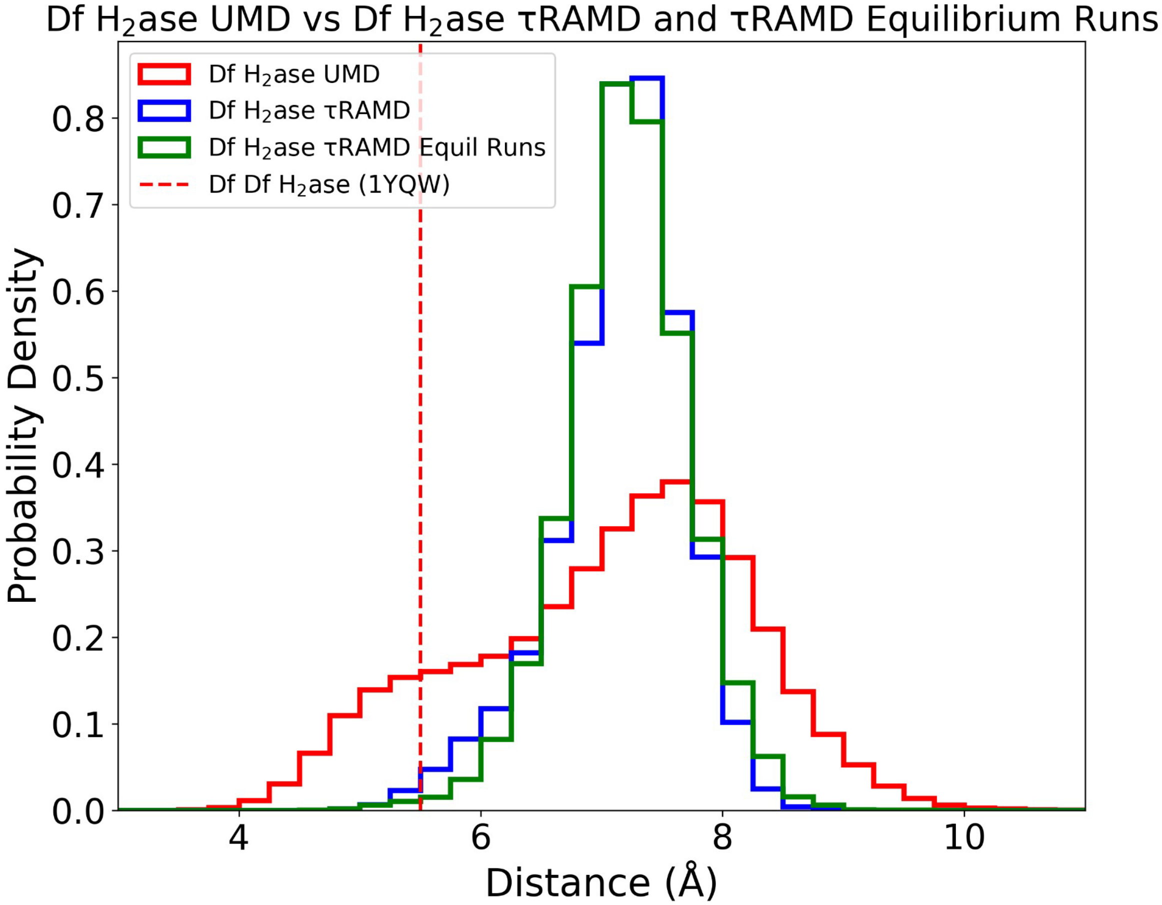

Figure S10. Probability density of lowest distance values between the bottleneck residues, V74 and L122, in UMD simulations of Df hydrogenase (75 replicas of 250 ns for H_2 binding and unbinding), in τRAMD simulations of Df hydrogenase (75 short replicas for H_2 unbinding) and in the equilibrium simulations performed prior to the τRAMD simulations (one trajectory of 50 ns, 5 trajectories of 20 ns, unbiased MD simulations with positional restraints to keep H_2 in the bound state). The distance value in the crystallographic structure is shown as a traced line.

Comment 3:

Since here unbiased MDs which are computationally expensive and for the minimal H2 molecules were compared, then we may consider that for exit pathways τRAMD works for any system?

Answer 3:

Based on the path probabilities we obtained with unbiased MD simulations and τRAMD, we observed that τRAMD can identify the most probable pathways for H_2 dissociation from hydrogenases. We expected this observation to be true for similar systems, where large

protein conformational changes are not required for ligand dissociation. We added a few sentences about it in the conclusion:
"We also compared the path probabilities from this work using UMD with the ones obtained in our previous works using τRAMD, and found that τRAMD can identify the most probable pathways for H_2 dissociation from hydrogenases. We expect this observation to be true for other systems, as long as large protein conformational changes are not required for ligand dissociation."

Technical part

Comment 4:

Why the authors used for the protein ff99sb and not an earlier version? Even if they used it in earlier versions succesfully why they did not try a comparison eg with ff19sb? I do not ask to run that huge work again, nut at least add a comment either in main text or SI.

Answer 4:

The reason to use the Amber ff99SB force field, instead of a newer version, is that the τRAMD simulations from our previous works have been performed with the Amber ff99SB force field, and we wanted to use the same force field for the unbiased MD simulations to make a proper comparison between the results from τRAMD simulations and unbiased MD simulations. We have plans to test and benchmark protein-ligand binding in hydrogenases using newer versions of the AMBER force field. The following text has been added to the method section in the main text:
"We kept the force field and, when possible, all of the parameters the same as the simulations performed previously with τRAMD for Df hydrogenase and Mdg hydrogenase[13,35] to make a fair comparison between results from τRAMD simulations and unbiased MD simulations."

Comment 5:

Why the authors performed their simulations at 40 oC?

Answer 5:

We performed simulations at 40°C to match the conditions of the experiments which were used to measure the Michaelis constant (K_m) for the reaction between H_2 and Df hydrogenase. This K_m value was used to estimate the experimental k_{on} value. A few sentences were added to the method section in the supporting information for clarification: "The MD simulations were performed at 313 K because the experiments to measure the Michaelis constant (K_m) for the reaction between H_2 and Df hydrogenase have been carried out at this temperature[5]. This K_m value was used to calculate the experimental k_{on} value."

Comment 6:

Did they perform the simulations at gpus? This should be noted in the paper. How long as regards real time the MDs take to reach a completion?

Answer 6:

We performed simulations using GPUs. Considering a performance of 40 ns/day, it took about 7 days to finish a 250 ns UMD simulation on one node on the HPC cluster we used. The following sentence was added to the methods section in the main text for clarification: "The MD simulations have been performed using two types of GPUs, Nvidia 1080 and 2080, on multiple nodes available at the High Performance Computer (HPC) of the Technical University of Berlin, and the performance for this system size, with 110,000 atoms, was roughly 40 ns/day on average."

Reviewer: 2

Recommendation: This paper is probably publishable, but major revision is needed; I do not need to see future revisions.

Major advance reported in the paper: The authors have used the process of H2 binding to the hydrogenase enzyme to demonstrate how unbiased simulations can be utilized to study the (un)binding kinetics and pathways of fast binding ligands. They also show that the unbinding pathways sampled using biased sampling methods, such as tau-RAMD, agree well with the unbiased results.

Immediate significance of this advance: This is a significant discovery, as it demonstrates the feasibility of using computationally tractable short molecular simulations to study the kinetics of complex biomolecular systems of physiological relevance. There is a dire need for efficient computational methods to screen drug candidates based on unbinding kinetics, and this paper shows that short unbiased MD simulations can be used in that regard. However, the scope of this work is somewhat limited by the fact that the authors only studied one simple fast-binding ligand, hydrogen, and did not show how their approach can be scaled to ligands with longer residence times.

Technical Suggestions: This paper can be published after addressing the following comments and concerns.

Comment 1:

It is unclear why these two specific hydrogenases were chosen for this work, rather than a pharmaceutically relevant system like the FKBP protein, where the conclusions may be more transferable to other biological phenomena.

Answer 1:

We chose these two specific [NiFe] hydrogenases because, as stated in the manuscript, binding rates for the substrate, H_2, are fast and it is therefore feasible to capture many binding events using UMD simulations. Additionally, in previous works we simulated the unbinding pathways of H_2 with an enhanced sampling method, τRAMD, and we wanted to compare the results from the two methods to test the reliability of τRAMD in sampling the most probable unbinding paths.

We agree with the reviewer that it would be interesting to perform UMD simulations using a pharmaceutically relevant system, and we are currently working in this direction. In the past, we considered FKBP as a target protein, but for FKBP-ligand complexes there are no experimental kinetic rates available, only experimental dissociation constants (K_d), which makes it challenging to benchmark UMD simulations and judge if enough events were captured to properly characterize binding. We are currently looking for other pharmaceutically relevant systems with experimental kinetic rates available, and for which it is feasible to capture a reasonable number of binding events using UMD simulations.

Comment 2:

It should be mentioned in the text that many short simulations, totaling 40 microseconds, were performed instead of one long unbiased simulation. It should also be mentioned that equilibrium properties should not be estimated directly from this ensemble of disconnected simulations.

Answer 2:

The text has been changed to highlight the fact that we performed 75 replicas of 250 ns to get 18.75 μs of UMD simulation for each hydrogenase, and to mention that equilibrium properties should not be directly estimated from this ensemble of simulations.

The following changes were made in the main text:

"In this work, we propose as a data set to benchmark the pathway probabilities obtained from enhanced sampling methods, the pathway probabilities for binding and unbinding of H_2 to and from two different [NiFe] hydrogenases, the hydrogenase from *Desulfovibrio fructosovorans* (Df hydrogenase) and the hydrogenase from *Megalodesulfovibrio gigas* (Mdg hydrogenase), obtained from a total of 18.75 μs of UMD simulations (75 replicas of 250 ns) for each hydrogenase."

"Equilibrium properties such as free energy landscapes (FEL) should not be directly computed from an ensemble of short independent simulations. Therefore, we constructed a Markov state

model (MSM; details in the supporting information, Figures S3-S8) and used it to compute a reweighted FEL (Figure 4B)."

Comment 3:

The authors mention observing both binding and unbinding events, but report only the kon from their computational study. The koff estimation from simulation, along with its agreement with the experimental values, should be provided in the main text.

Answer 3:

In the publication used by us as a reference for experimental data (Liebgott et al.[5]), there is only the experimental Michaelis constant (K_m) for the reaction between H_2 and Df hydrogenase reported. This K_m value was used to calculate the experimental k_{on} value (Equations 1-3 in the supporting information). That is why we could only compare experimental and computational k_{on} values. The experimental catalytic rate for the substrate, H_2, is expected to be much faster than the k_{off} value[5,6] and, therefore, the k_{off} value was not reported in the publication mentioned. The k_{off} value computed from UMD simulations was added to the main text.

"The computational k_{off} value was calculated to be $2.17 * 10^8$ s^{-1}, but there is no experimental k_{off} value reported for comparison."

Comment 4:

Observing low binding/unbinding events with O2 and CO may indicate that they bind strongly or their kinetics is slower. As the statistics are low, it may not be possible to be quantitative, but this qualitative effect should not be ignored, and their pathways should also be reported.

Answer 4:

Following the reviewer's suggestion, information about the pathways for CO and O_2 (un)binding have been added to the supporting information (Table S3).

While performing a more detailed analysis of the binding events for O_2 and CO, we found that the actual number of binding and unbinding events was lower than what was reported in the original version of the manuscript. The text was corrected accordingly.

"We performed the same UMD simulations for the inhibitors O_2 and CO binding to Df hydrogenase, as we did for the substrate H_2. However, the number of (un)binding events obtained were low, 4 and 2 binding and 4 and 1 unbinding events for O_2 and CO, respectively, even with double the amount of simulation time for CO (37.5 µs). The pathways for O_2 and CO can be found in Table S3."

Table S3. O_2 and CO binding and unbinding events captured in 75 and 150 independent UMD simulations (250 ns length), respectively, for Df hydrogenase.

Molecule	Replica #	Tunnels in[a]	First Passage Time - Binding[b] (ps)	Tunnels out[c]	Time of the unbound state[d] (ps)	First Passage Time - Unbinding[e] (ps)
O_2	29	T2	7460	T2	45530	38070
O_2	4	T1	236550	T1	246800	10250
O_2	50	T1	188890	T8	230350	41460
O_2	62	T2	62400	T1	72410	10010
CO	27	T1	161400	T1	211270	49870
CO	54	T1	196670	NA[f]	NA[f]	NA[f]

a) "Tunnel in" refers to the tunnel that the H_2 molecule used for binding.

b) "First Passage Time - Binding (ps)" refers to the time in the simulation when the H_2 molecule reached the bound state. The start time was the start of the simulation.

c) "Tunnel out" refers to the tunnel that the H_2 molecule used for unbinding.

d) "Time of the unbound state (ps)" refers to the time in the simulation when the unbound state was achieved.

e) "First Passage Time - Unbinding (ps)" is the time it took the H_2 molecule to reach the unbound state, starting from the bound state.

f) NA means 'Not Available'. In some cases, especially near the end of the simulation, only binding events were captured and no unbinding events could be achieved.

Comment 5:

How the pathways of binding were classified as symmetric using the chi-square test is not clear. How is it possible to characterize a pathway using a single distance? How is the distribution of pathways computed?

Answer 5:

Regarding the characterization of the pathways, we first identified tunnels for gas diffusion in the crystallographic structure using CAVER, following our previous work[1]. Then, we attributed the (un)binding events (identified using the distance criteria) to specific tunnels by visual inspection, based on the matching of entry (for binding events) and exit points (for unbinding events) between tunnels and (un)binding events. Note that we use the term "tunnel" to refer to voids in the crystal structure that may be used for (un)binding, and the term "pathway" to refer to a tunnel that is effectively used for (un)binding during the UMD simulation. The procedure is described in the main text, which we edited to improve clarity:

"We mapped the tunnels for gas diffusion in the crystallographic structures of both Df and Mdg hydrogenases using CAVER 3.0 (Figure 2) to later map the tunnels to the (un)binding events identified in UMD simulations, following our previous works[13,35]. We found that the tunnels are

similar for the different enzymes, except for the fact that T9 in Df hydrogenase is not present in Mdg hydrogenase. There are also changes in the tunnels T3 and T8 in the Mdg hydrogenase, which have common parts with T1, in contrast to T3 and T8 in Df hydrogenase, which are independent of T1. The binding and unbinding events obtained from UMD for Df and Mdg hydrogenase were identified following the definitions of bound and unbound states above, and manually assigned to the tunnels identified (Figure 3, Table S4). The assignment was based on the matching of entry (for binding events) and exit points (for unbinding events) between (un)binding events and tunnels. We use the term "pathway" to refer to a tunnel used for (un)binding in the UMD simulations."

The distribution of pathways was computed by counting the number of (un)binding events that followed a particular tunnel. The number of events and associated probabilities can be seen in table S4:

Table S4. Percentages (number of events) of tunnels used for binding and unbinding in Df and Mdg hydrogenases (raw data of Figure 3).

	**T1**	**T2**	**T3**	**T4**	**T5**	**T6**	**T7**	**T8**	**T9**
Df hydrogenase - Binding	25.5% (11)	46.5% (20)	11.6% (5)	11.6% (5)	0.0% (0)	0.0% (0)	0.0% (0)	4.6% (2)	0.0% (0)
Df hydrogenase - Unbinding	31.7% (13)	24.3% (10)	4.8% (2)	9.7% (4)	7.3% (3)	0.0% (0)	2.4% (1)	19.5% (8)	0.0% (0)
Mdg hydrogenase - Binding	22.2% (22)	47.4% (47)	13.1% (13)	2.0% (2)	0.0% (0)	0.0% (0)	0.0% (0)	15.1% (15)	0.0% (0)
Mdg hydrogenase - Unbinding	23.4% (23)	57.1% (56)	9.1% (9)	1.0% (1)	3.0% (3)	0.0% (0)	0.0% (0)	6.1% (6)	0.0% (0)

Using the number of events for each tunnel reported in table S4, we performed the chi-square test to assess whether there was symmetry of the binding and unbinding pathways for the two hydrogenases or, in other words, if the pathway probabilities for binding and unbinding were the same. More specifically, the chi-square test assessed whether the distribution of events in different categorical variables (pathways) was the same in the two

different groups (binding and unbinding events). We modified the main text to improve clarity:

"Next, we tested whether there was symmetry of the binding and unbinding pathways for the two hydrogenases or, in other words, if the pathway probabilities for binding and unbinding were the same. Pathway symmetry is expected for equilibrium processes, based on the principle of detailed balance or microscopic reversibility proposed by Boltzmann[42]. A chi-square test was conducted, and the p-values obtained were 0.053 and 0.121 for Df hydrogenase and Mdg hydrogenase, respectively, which indicates that the differences in pathway usage between binding and unbinding events in each hydrogenase are not statistically significant (using a p-value of 0.05 as a threshold). The p-value for Df hydrogenase is near the threshold, and this can be traced to the differences in the populations of paths T5 and T8 (Tables S5 and S6). Paths T5 and T8 have lower probability in binding events. Such differences can be the result of a limited number of (un)binding events."

Comment 6:

Is the free energy landscape in Figure 4b computed using Markov state models (MSM) or some other method? Otherwise, how are multiple disconnected trajectories included together?

Answer 6:

Figure 4b is a reweighted free energy landscape computed using a MSM that was not reported in the original version of the manuscript. In the revised version of the manuscript, we improved the description of Figure 4b (to highlight that the free energy landscape was computed using MSM) and included more information of the methods and results obtained with MSM in the supporting information.

In the main text:

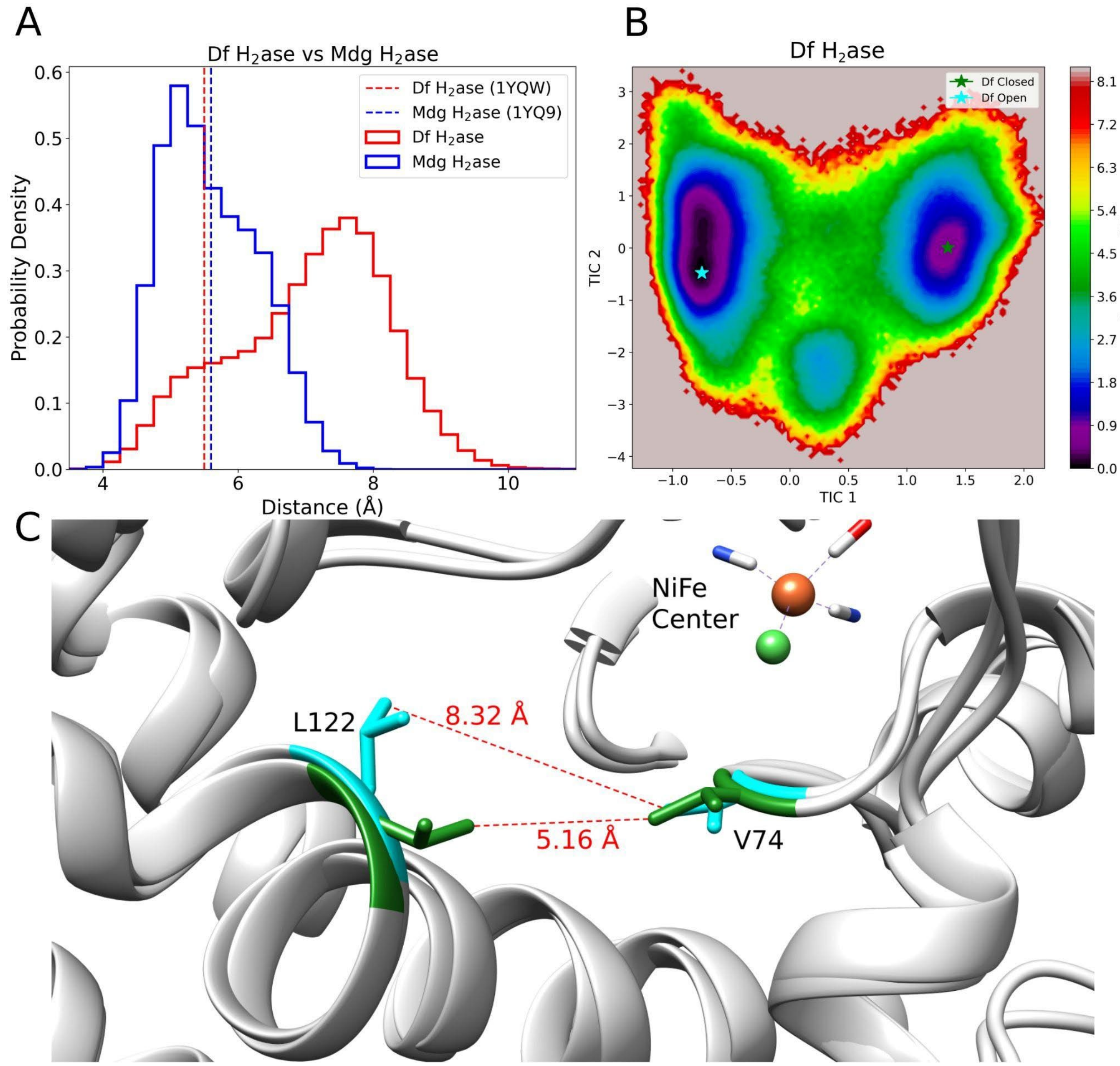

Figure 4. Dual-state bottleneck in Df hydrogenase (Df H_2ase) observed in unbiased molecular dynamics simulations. A) Probability density of lowest distance values between residues V74 and L122 in Df hydrogenase and Mdg hydrogenase (Mdg Df H_2ase). The distance values in the crystallographic structures are shown as traced lines. B) Free energy landscape of the V74-L122 bottleneck in Df hydrogenase computed using a Markov state model (details in the supporting information). Two main states were identified, open and closed. C) Representative snapshots of Df hydrogenase in the open (cyan residues) and closed states (green residues) of the V74-L122 bottleneck. The location of the snapshots in the free energy landscape in panel B is indicated with green and cyan stars.

In the supporting information:

"In order to find and characterize the states of the main bottleneck and build free energy landscapes, we constructed MSMs for Df hydrogenase and Mdg hydrogenase. This section

describes the methods and results obtained for Df hydrogenase. The MSM was built using pyemma 2.5.2 program[31] and, as features, we used the pairwise distances between all heavy atoms of the V74 and L122 residues during the entire UMD simulations, which consist of 75 concatenated independent simulations of 250 ns each (37.5 µs in total). The high dimensional data was reduced using TICA with a lag time of 1 ns and 4 dimensions. Then, the kmeans method was used to cluster the configurations into 200 microstates. After the clustering, we obtained the discretized trajectories and the Markovian behavior was examined via the implied timescales plot (Figure S3). As shown in the figure, the plateau at longer timescales indicates Markovian behavior. After that we performed dimensionality reduction, clustering and obtained some discretized trajectories, and a MSM model with a lag time of 4 ns was constructed. Then, the Chapman-Kolmogorov test was used to validate the MSM model (Figure S4). Next, based on the implied timescales plot, the microstates were lumped into 4 main macrostates using the PCCA method[34] (Figure S5; the black and red dots represent the open and closed states of the bottleneck, respectively). The populations obtained for macrostates 1 (open state), 2 (closed state), 3 and 4 were 63.8%, 30.6%, 4.4% and 1.2%, respectively (Figure S6). The populations were calculated by summing the stationary probabilities of microstates in each macrostate. In the main text, we focused on the most populated states, open and closed states. Then, the rate matrix was generated in order to describe continuous-time transition rates between macrostates using the equation below:

$$K = \frac{1}{\tau}(P - I) \quad (6)$$

In which **P** is the discrete-time transition probability matrix, **I** is the identity matrix, **τ** is the MSM lag time and **K** gives the rate from one state to another. Moreover, for the MSM macrostates, the MFPT can be calculated using equation 7. The MFPT can be interpreted as the waiting time needed to go from one state to the other. Figures S7 and S8 show the macrostate transition network with the corresponding waiting times.

A jupyter notebook containing all the code for the MSM model building is available at (https://github.com/FarzinSohraby/SI-H2ase-UMD).

$$MFPT_{a \to b} \approx \frac{1}{k_{a \to b}} \quad (7)$$

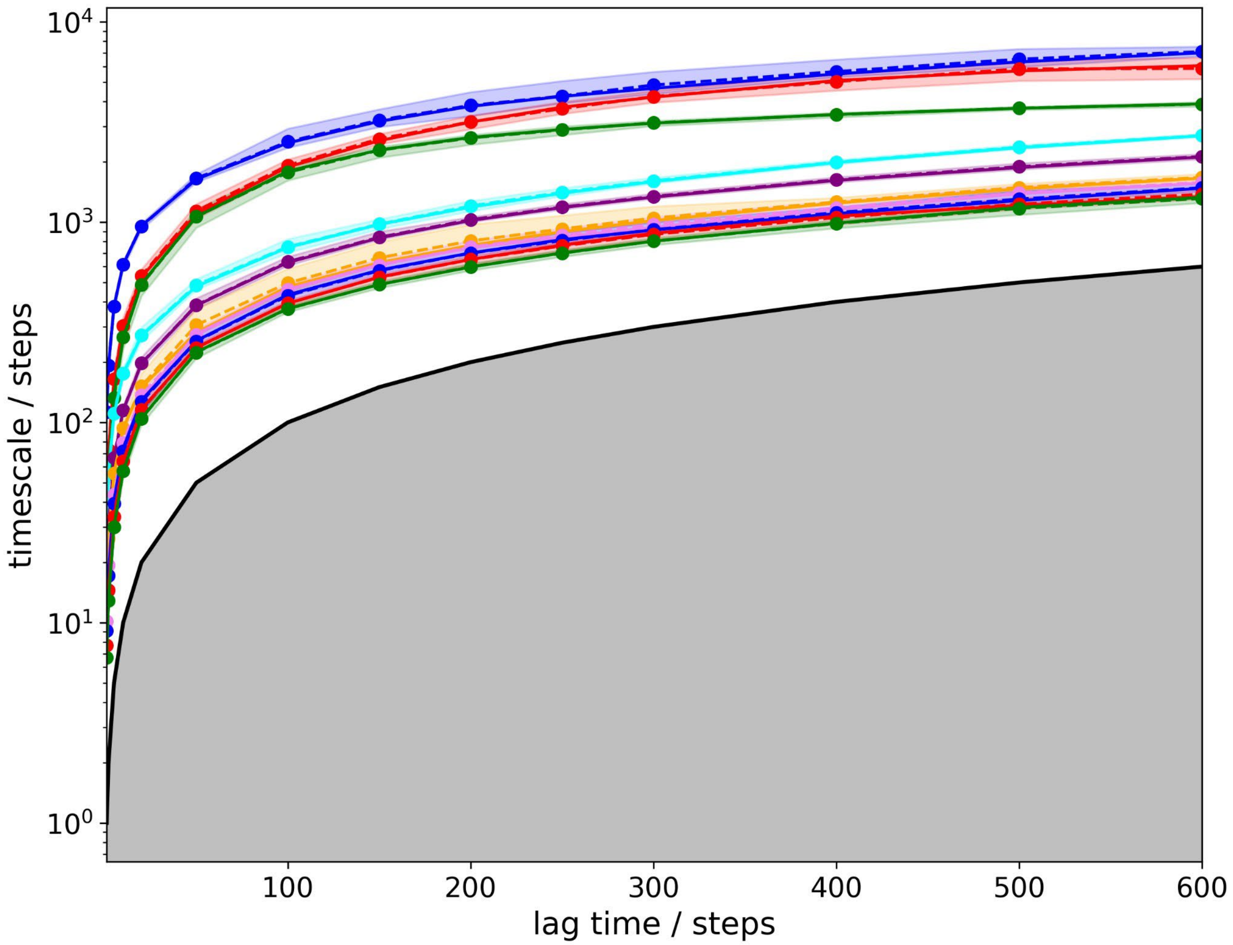

Figure S3. Implied timescales (ITS) analysis of the discretized trajectories using a Bayesian error estimate. Each colored line represents one of the top 10 slowest dynamical processes (ITS) extracted from the transition matrix at each lag time. Different colors distinguish between different timescales (eigenvalues of the MSM transition matrix). Solid lines represent the median estimated ITS for each lag time. Shaded areas (or if applicable, error bars/traced lines) reflect the Bayesian uncertainty (posterior confidence intervals) over the implied timescale estimates from bootstrapped MSM samples. The gray shaded region below the black curve indicates where the estimated timescales fall below the lag time.

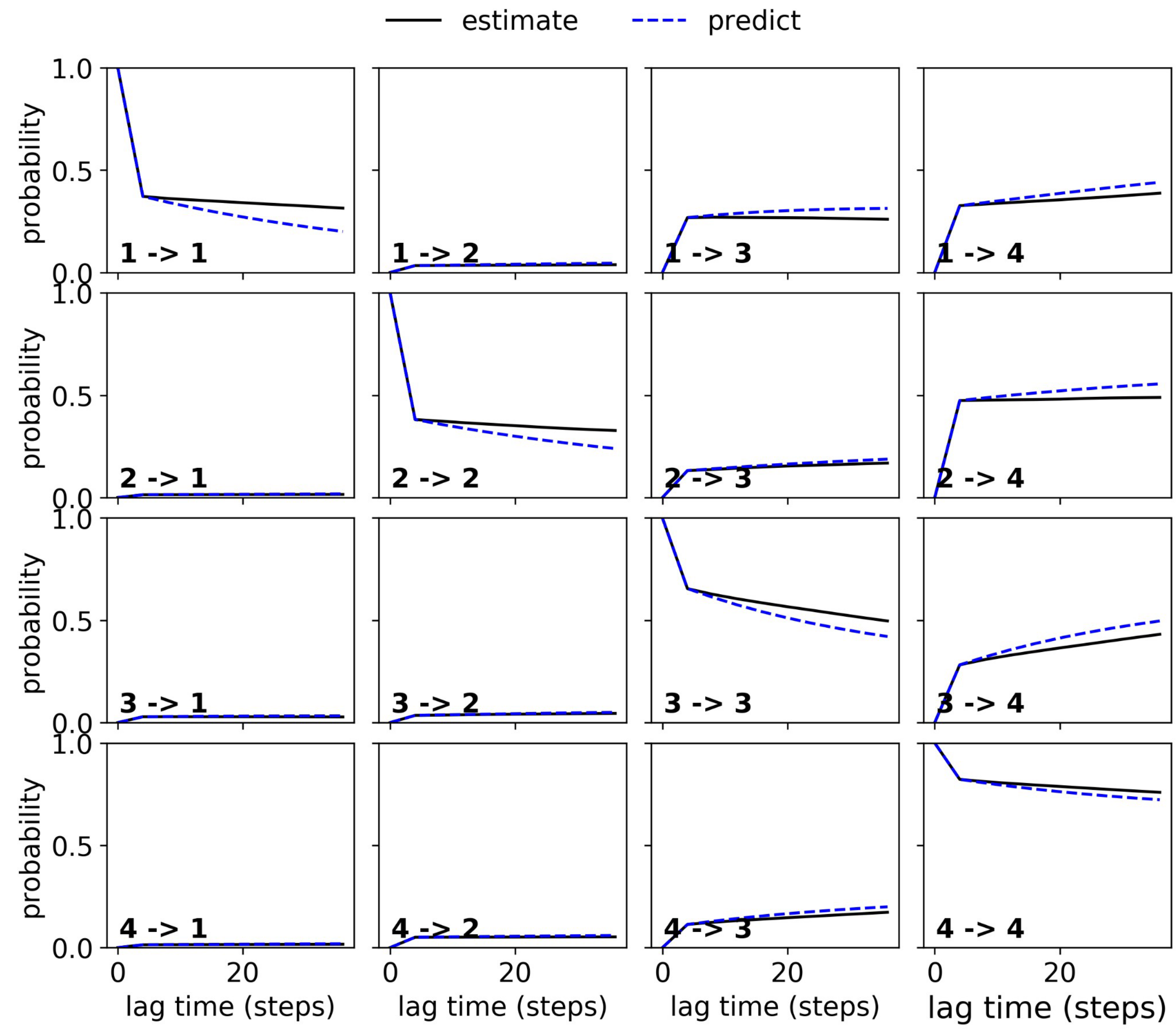

Figure S4. Chapman-Kolmogorov test for the constructed MSM model using 4 macrostates.

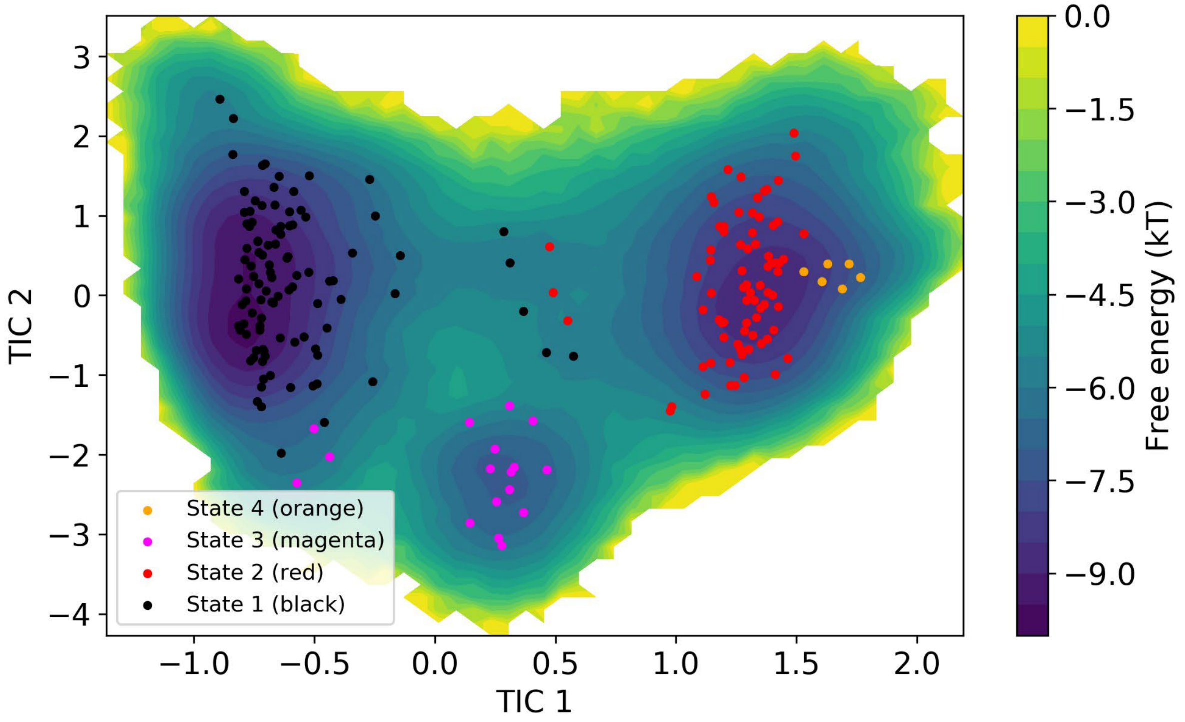

Figure S5. Free energy landscape with positions of microstates and macrostates obtained from MSM. Red and black dots represent the closed and open states. Each dot represents a microstate and is colored by its assigned macrostate.

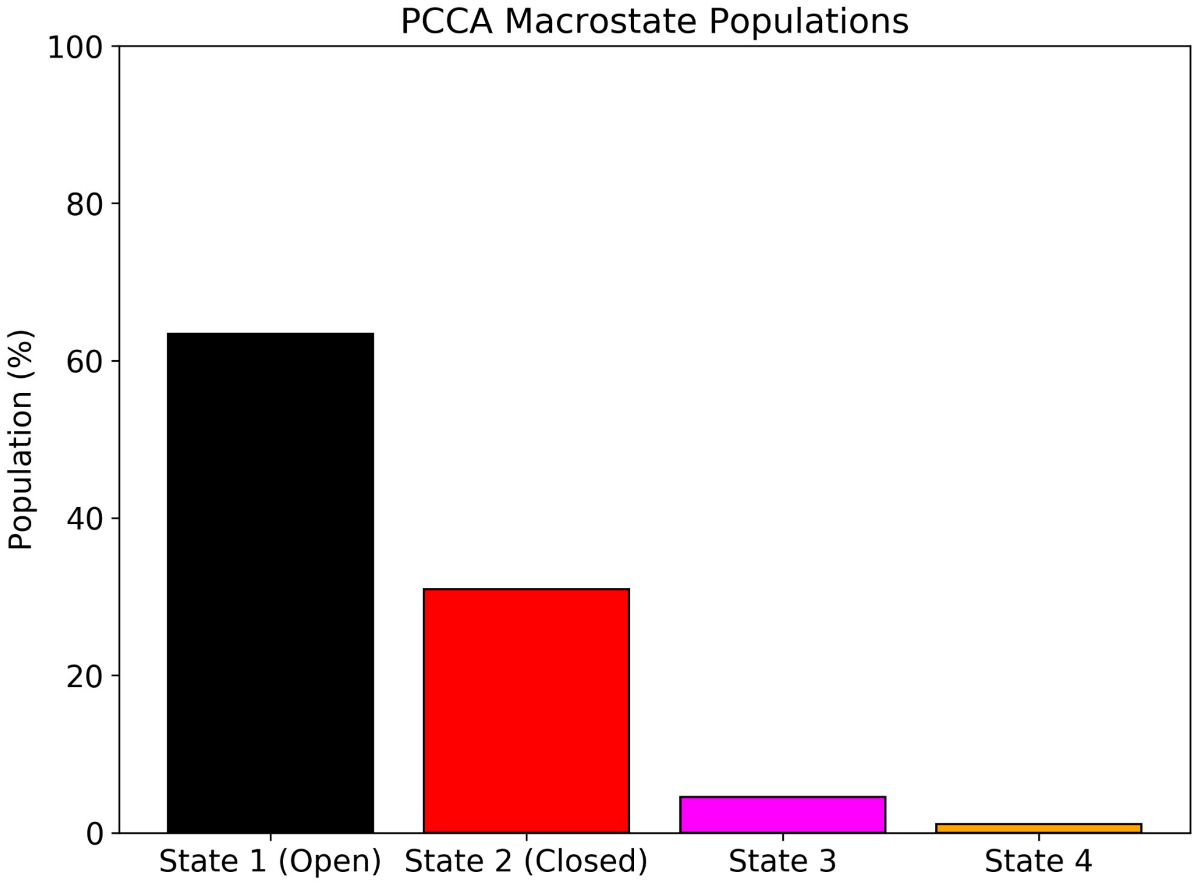

Figure S6. Populations of the 4 macrostates identified in MSM.

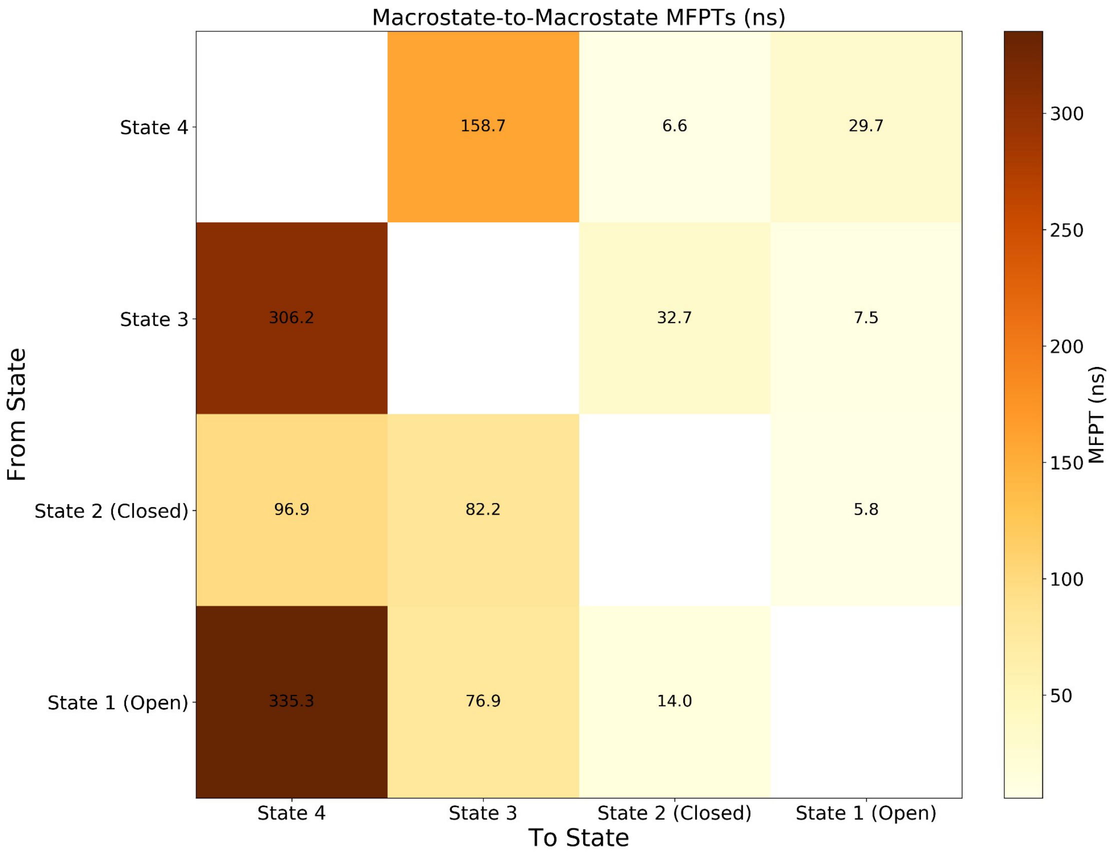

Figure S7. Matrix of the mean first passage times (MFPTs, in ns) between the four macrostates obtained from MSM.

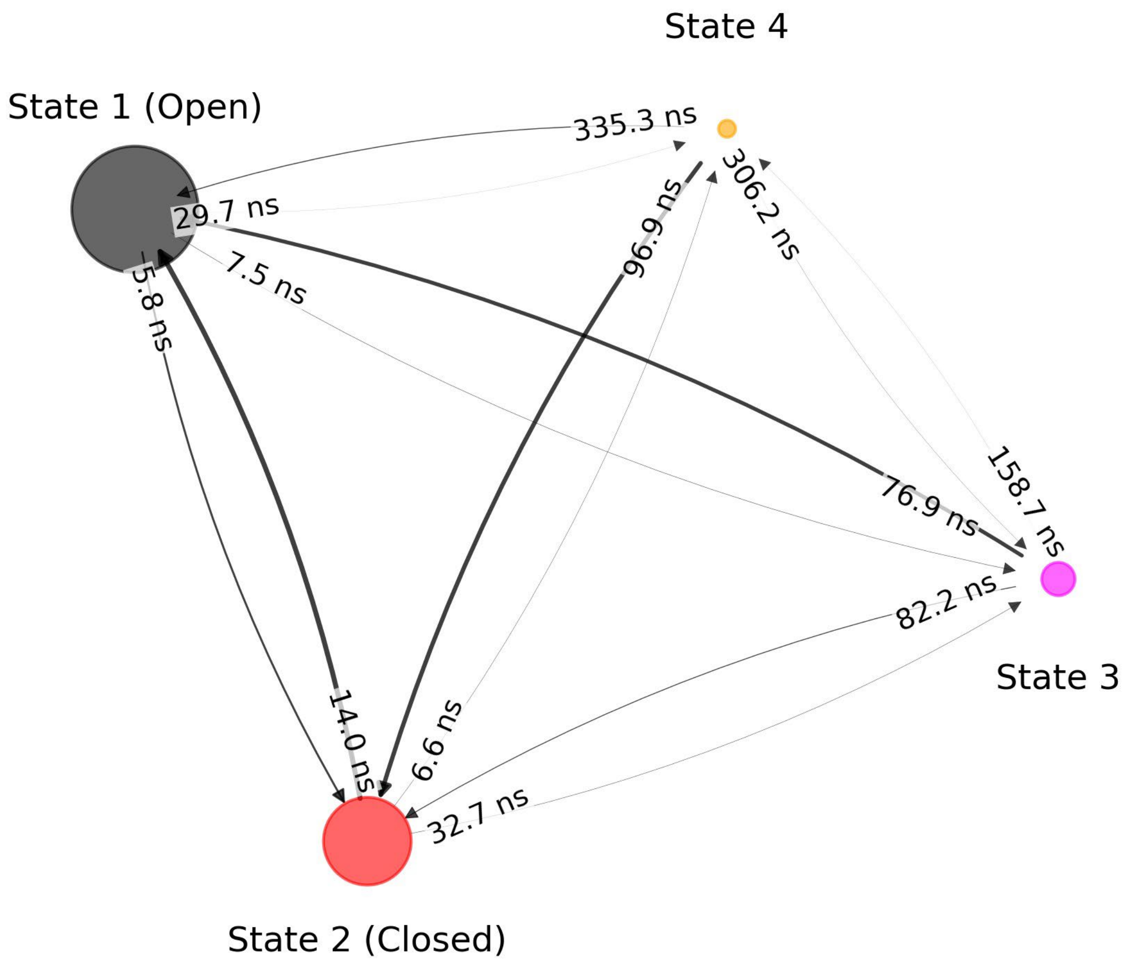

Figure S8. Transition network of the 4 macrostates obtained from MSM. The sizes of the nodes of the macrostates represent their population in the MSM.

Comment 7:

Minor style-related comment: The authors should just use the word "hydrogenase" instead of H2ase. It does not reduce the word count and makes it difficult to read.

Answer 7:

Following the reviewer's suggestion, the word "H_2ase" has been replaced by "hydrogenase" throughout the text of the manuscript and supporting information.

References

(1) Sohraby, F.; Nunes-Alves, A. Characterization of the Bottlenecks and Pathways for Inhibitor Dissociation from [NiFe] Hydrogenase. *J. Chem. Inf. Model.* **2024**, *64* (10), 4193–4203. https://doi.org/10.1021/acs.jcim.4c00187.

(2) Sohraby, F.; Guo, J.-Y.; Nunes-Alves, A. PathInHydro, a Set of Machine Learning Models to Identify Unbinding Pathways of Gas Molecules in [NiFe] Hydrogenases. *J. Chem. Inf. Model.* **2025**, *65* (2), 589–602. https://doi.org/10.1021/acs.jcim.4c01656.

(3) Wang, P.; Blumberger, J. Mechanistic Insight into the Blocking of CO Diffusion in [NiFe]-Hydrogenase Mutants through Multiscale Simulation. *Proc. Natl. Acad. Sci.* **2012**, *109* (17), 6399–6404. https://doi.org/10.1073/pnas.1121176109.

(4) Wang, P.; Best, R. B.; Blumberger, J. Multiscale Simulation Reveals Multiple Pathways for H2 and O2 Transport in a [NiFe]-Hydrogenase. *J. Am. Chem. Soc.* **2011**, *133* (10), 3548–3556. https://doi.org/10.1021/ja109712q.

(5) Liebgott, P.-P.; Leroux, F.; Burlat, B.; Dementin, S.; Baffert, C.; Lautier, T.; Fourmond, V.; Ceccaldi, P.; Cavazza, C.; Meynial-Salles, I.; Soucaille, P.; Fontecilla-Camps, J. C.; Guigliarelli, B.; Bertrand, P.; Rousset, M.; Léger, C. Relating Diffusion along the Substrate Tunnel and Oxygen Sensitivity in Hydrogenase. *Nat. Chem. Biol.* **2010**, *6* (1), 63–70. https://doi.org/10.1038/nchembio.276.

(6) Liebgott, P.-P.; Dementin, S.; Léger, C.; Rousset, M. Towards Engineering O_2-Tolerance in [Ni–Fe] Hydrogenases. *Energy Env. Sci* **2011**, *4* (1), 33–41. https://doi.org/10.1039/C0EE00093K.

(7) Lewis, G. N. A New Principle of Equilibrium. *Proc. Natl. Acad. Sci.* **1925**, *11* (3), 179–183. https://doi.org/10.1073/pnas.11.3.179.

(8) Scherer, M. K.; Trendelkamp-Schroer, B.; Paul, F.; Pérez-Hernández, G.; Hoffmann, M.; Plattner, N.; Wehmeyer, C.; Prinz, J.-H.; Noé, F. PyEMMA 2: A Software Package for Estimation, Validation, and Analysis of Markov Models. *J. Chem. Theory Comput.* **2015**, *11* (11), 5525–5542. https://doi.org/10.1021/acs.jctc.5b00743.

(9) Schütte, C.; Fischer, A.; Huisinga, W.; Deuflhard, P. A Direct Approach to Conformational Dynamics Based on Hybrid Monte Carlo. *J. Comput. Phys.* **1999**, *151* (1), 146–168. https://doi.org/10.1006/jcph.1999.6231.
